# Influence of diurnal variations on cognitive coordination and misunderstanding in elite male handball players

**DOI:** 10.7717/peerj.20370

**Published:** 2026-01-15

**Authors:** Sana Essid, Hatem Ghouili, Yassine Negra, Tony D. Myers, Halil ibrahim Ceylan, Valentina Stefanica, Moktar Chtara, Haifa Jemili, Hamdi Chtourou, Nizar Souissi, Ismail Dergaa

**Affiliations:** 1 Research Unit “Physical Activity, Sport and Health” (UR18JS01), National Observatory of Sports, Tunis, Tunisia; 2 Research Laboratory (LR23JS01) Sport Performance, Health & Society, High Institute of Sport and Physical Education, Ksar-Saïd, Manouba University, Manouba, Tunisia; 3 High Institute of Sport and Physical Education of Kef, University of Jendouba, El Kef, Tunisia; 4 Sport and Health, Newman University, Birmingham, United Kingdom; 5 Physical Education of Sports Teaching Department, Faculty of Sports Sciences, Ataturk University, Erzurum, Turkey; 6 Department of Physical Education and Sport, Faculty of Sciences, Physical Education and Informatics, Pitesti University Center, University Politehnica of Bucharest, Pitesti, Romania; 7 Unit Head of Scientific Research and Studies, Sharjah Women’s Sports United Arab Emirates, Sharjah, United Arab Emirates; 8 Research Laboratory Education, Motricité, Sport et Santé (EM2S) LR19JS01, High Institute of Sport and Physical Education of Sfax, University of Sfax, Sfax, Tunisia; 9 Primary Health Care Corporation (PHCC), Doha, Qatar; 10 High Institute of Sport and Physical Education of Ksar Saïd, University of Manouba, Manouba, Tunisia

**Keywords:** Athletic performance, Chronobiology, Cognitive processes, Coordination, Decision making, Interpersonal communication

## Abstract

This study examined how the time of day influences cognitive coordination in elite male handball players. We investigated misunderstandings and contradictions during cognitive exchanges to identify optimal times for shared understanding and collective performance. Six elite male handball players (age 17.5 ± 0.2 years) from Tunisia’s first division participated in simulated matches at three different time points: (10h00, 14h00, and 18h00). We analyzed cognitive coordination using audiovisual recordings and self-confrontation interviews, applying the Recognition-Primed Decision (RPD) model. Results revealed significantly higher frequencies of contradictory and misunderstanding coordination forms during morning sessions compared to afternoon and evening sessions across all cognitive components (actions, relevant cues, goals, expectations). These findings suggest that circadian factors influence team cognitive coordination, with the afternoon and evening periods being optimal for collective performance, which requires sophisticated coordination and a shared understanding.

## Introduction

In various team sports, including handball and soccer, the role of cognitive content in certain situations (such as defense and attack) has been identified as a decisive factor for success. This assertion is supported by researchers such as Bossard et al. (2010), Bourbousson, Feigean & Seiler (2019), and De Keukelaere et al. (2013), who have emphasized the importance of situational cognitive strategies in achieving superior team outcomes. Recognizing these strategic cognitive elements forms the basis for a deeper examination of how teams collectively navigate and interpret complex game situations. Within this collective framework, the concept of situational awareness gains prominence. Furthermore, collective situational awareness, which refers to team members’ shared understanding and processing of situational dynamics, has been the subject of extensive research (Cooke & Gorman, 2006; Klein, 2008; Salmon et al., 2017). The psychological mechanism behind this phenomenon, collective cognition, is thought to play a central role in the overall performance of sports teams (Salas et al., 2008; Gray et al., 2017). As defined by Cooke et al. (2013), collective cognition refers to the cognitive activities that occur at the team level, capturing the shared thought processes that facilitate coordinated action and strategic decision-making in team sports. However, the complex interplay between these cognitive processes and the physiological underpinnings regulated by circadian rhythms remains an area that has received little research attention.

Cognitive abilities in elite handball encompass executive attention, working memory capacity, cognitive flexibility, inhibitory control, and processing speed, which collectively determine situational awareness and decision-making effectiveness under competitive pressure (Blecharz et al., 2022; Scharfen & Memmert, 2019; Trecroci et al., 2021). These abilities manifest through sophisticated pattern recognition, anticipatory skills, and spatial–temporal processing capabilities that distinguish elite from sub-elite performers (Araújo, Davids & Renshaw, 2020; Starkes & Ericsson, 2003). Research demonstrates that handball players exhibit position-specific cognitive profiles, with significant variations in selective attention, peripheral perception, and reaction time capabilities across different playing roles (Blecharz et al., 2022).

Psychomotor abilities integrate cognitive processing with motor execution through reaction time, movement time, choice reaction time, and psychomotor vigilance mechanisms (Przednowek et al., 2019). Elite handball players demonstrate superior psychomotor capabilities compared to recreational players, particularly in complex decision-making scenarios requiring rapid stimulus–response coordination under competitive pressure (Tenenbaum, Basevitch & Gutierrez, 2015). These abilities are fundamental for effective team coordination, as they enable players to rapidly process tactical information and execute coordinated responses within the temporal constraints of competitive play, forming the foundation for collective cognitive performance.

Decision-making in handball involves rapid situation assessment, option generation, evaluation, and action selection occurring within milliseconds during dynamic competitive play (Starkes & Ericsson, 2003; Tenenbaum, Basevitch & Gutierrez, 2015). These processes integrate multiple cognitive components and are significantly influenced by expertise level, positional specialization, and temporal factors affecting cognitive readiness and information processing efficiency. Understanding these individual cognitive foundations is crucial for examining how circadian variations impact collective cognitive coordination and team performance outcomes.

Recent advances in chronopsychology have demonstrated significant circadian variations in executive function, cognitive flexibility, attention control, and working memory capacity, with peak performance typically occurring during individual’s circadian preference periods (Schmidt et al., 2021; Facer-Childs et al., 2021). Contemporary research reveals complex interactions between chronotype, circadian timing, and cognitive performance in athletic contexts, with significant implications for team coordination and collective decision-making processes (Vitale et al., 2022). These findings provide a crucial theoretical foundation for understanding how individual circadian variations aggregate to influence team-level cognitive coordination patterns.

Recent studies conducted by Munnilari et al. (2024) in chronopsychology, which examine biological rhythms and their impact on behavior and cognitive functions, reveal fluctuations in performance variables related to cognitive abilities. These reach a peak at specific times of the day. Circadian rhythms, in particular, affect alertness, mood, and cognitive and physical performance, and regulate numerous physiological and psychological processes, thereby influencing athletes’ ability to compete effectively. Ayala et al. (2021) consolidate this line of thinking in their study, specifying that most parameters governing athletic performance fluctuate depending on the time of day and peak in the evening, thus influencing the interaction between team members, which is a determining factor in team sports performance (Vogel & Schack, 2023). Although these cognitive processes are recognized, the influence of physiological and environmental factors, particularly circadian rhythms, on team cognition and performance has not been thoroughly explored. Circadian rhythms, which control a wide range of physiological processes, have been shown to significantly influence individual athletic performance, peaking in the late afternoon and early evening (Dergaa et al., 2019; Dergaa et al., 2020; Romdhani et al., 2021; Souissi et al., 2022). This time coincides with the natural peak of core body temperature and indicates a potential peak in cognitive and physical performance. Yet, the application of these findings to team performance, particularly about collective cognition and situational awareness, remains under-researched.

To complicate matters, there is evidence that time of day influences not only physical performance but also cognitive and mood-related aspects of athletes’ performance (Mhenni et al., 2017; Essid et al., 2022). This suggests a potential for optimizing team performance by synchronizing the circadian rhythms of team members and aligning their peak cognitive and physical performance functioning times.

Based on the proven importance of cognitive strategies in team sports, the under-researched influence of circadian rhythms on collective cognition and situational awareness, and the potential to optimize team performance through circadian synchronization, this study aimed to investigate how different times of day influence cognitive sharing, understanding, and coordination in elite male handball players.

This investigation builds upon our comprehensive psychological assessment of identical participants (Essid et al., 2022), which demonstrated significant circadian variations in negative mood states (anxiety, anger, confusion, depression, fatigue; all *p* < 0.05) and increased fatigue indices during morning hours (10h00) compared to afternoon and evening sessions. These psychometric findings provide essential physiological context for understanding the cofnitive coordination within established circadian frameworks.

## Materials and Methods

### Participants

Six elite male handball players (age = 17.5 ± 0.2 years, body mass = 78.7 ± 6.1 kg, height = 1.8 ± 0.03 m) voluntarily participated in the experimental procedure of this study. Participants were affiliated with a professional club competing in Tunisia’s first division handball league, representing the highest level of handball competition in Tunisia. These athletes maintained rigorous training schedules of six sessions per week (12–15 h total weekly training volume) and regularly participated in official championship matches against other first division teams. Several participants held youth national team representation, indicating elite-level expertise and competitive experience essential for examining sophisticated cognitive coordination dynamics. The competitive level ensures ecological validity for investigating cognitive coordination patterns relevant to high-performance handball contexts. All participants classified as “moderately evening” chronotype based on the circadian typology questionnaire by Horne & Ostberg (1976) were eliminated as a confounding variable, following established chronobiology protocols (Roenneberg, Wirz-Justice & Merrow, 2003). This methodological decision enhances internal validity by controlling for individual chronotype variations while enabling focused analysis of temporal effects on cognitive coordination. Although limiting generalizability to other chronotype populations, this homogeneity represents an appropriate experimental control for exploratory hypothesis-generating research.

Chronotype assessment employed the Horne-Östberg questionnaire, which remains the gold standard in circadian research due to its extensive validation across diverse populations, superior psychometric properties, and widespread contemporary use, facilitating comparison with existing literature (validated in over 50 languages with >3,000 recent citations). While acknowledging contemporary chronotype assessment tools, we employed this established instrument to ensure methodological consistency with chronobiology research protocols and to enable meaningful comparison with the extensive literature base on circadian rhythms.

The sleep times of these participants were determined using the calendar of Bastuji & Jouvet (1985) for one month. The estimated mean sleep duration was 7.5 h (±0.5). To ensure the confidentiality of the data, each participant was given a pseudonym related to their position on the pitch: right winger, right back, half center, pivot, left back, and left winger.

All experimental procedures and their associated potential risks were thoroughly explained to both participants and parents, with written informed consent obtained from participants’ parents or legal guardians before study commencement. Comprehensive consent procedures included a detailed explanation of research objectives, experimental methods, time commitments, potential risks, confidentiality measures, and voluntary participation rights. This study was conducted in accordance with the Declaration of Helsinki for human research ethics and received full approval from the Ethics Committee of the High Institute of Sport and Physical Education, Ksar-Saïd, Manouba University (Approval No. CPP: 104/2022). Additionally, the research adhered to all ethical and procedural standards recommended by the journal (Portaluppi, Smolensky & Touitou, 2010).

### Experimental procedure

Our intensive case study design (*n* = 6) represents a deliberate methodological choice, enabling a comprehensive analysis of position-specific cognitive coordination patterns through an extensive self-confrontation methodology. Building upon our previous study of eighteen handball players (Essid et al., 2022), the current investigation focused on six field positions to facilitate detailed analysis of cognitive coordination during offensive phases. The 13.5 h of verbalization data (45 min × 3 sessions × 6 players) provide unprecedented depth of qualitative evidence for examining cognitive coordination dynamics, aligning with established phenomenological research approaches in elite sports psychology where intensive data collection methods necessitate smaller samples to achieve analytical depth. Participants completed three training matches at three different times of the day (*i.e.,* 10h00, 14h00, and 18h00), with at least 48 h of recovery time between each session.

The order of match times was systematically randomized across participants using a complete randomized block design, with participants assigned to one of six possible testing sequences, ensuring balanced exposure to different temporal conditions. This randomization procedure controlled for potential order effects, learning confounds, and sequence bias that could systematically influence cognitive coordination patterns. Randomization was conducted using computer-generated random number sequences, with sequence assignment concealed until completion of baseline assessments.

Systematic recovery and learning control measures included: minimum 48-hour recovery periods between consecutive sessions to minimize fatigue confounds and ensure consistent physiological baseline conditions, learning effect control through comprehensive pre-testing familiarization sessions, varied offensive tactical scenarios across sessions maintaining cognitive challenge consistency, and systematic variation in opposing team strategies to prevent tactical adaptation while maintaining equivalent competitive difficulty across all temporal conditions.

Simulated matches were conducted under standardized competitive conditions employing complete 6-versus-6 opposition between teams of similar skill levels from the Tunisian first division handball league. Each session was implemented according to official handball rules, with certified referee supervision, regulation court dimensions and equipment, and controlled environmental conditions (temperature: 20−22 °C, standardized LED lighting systems). Opposition teams consisted of players from identical competitive levels, ensuring authentic tactical complexity and competitive intensity. Matches followed 2  ×  15-minute periods with official timing, implementing complete rule enforcement and authentic competitive pressure.

Competitive authenticity was enhanced through comprehensive environmental replication, including coaching staff presence that provided tactical instructions and performance feedback, teammate support from non-participating players that created an authentic social context, official scorekeeping and performance timing, and post-match analysis sessions consistent with competitive match protocols. Matches were conducted on participants’ home court, maintaining familiar environmental conditions while ensuring standardized opposition quality and competitive challenge across all temporal conditions.

Participants were asked to maintain their usual sleep habits, (*i.e.,* to sleep for at least 7,5 h per night) during the experimental period, to keep their normal level of physical activity and to refrain from any intensive activities on the day before each test session.

Comprehensive confounding variable control protocols included: (1) standardized nutritional guidelines with consistent pre-session meals (carbohydrate-rich breakfast 2 h before testing) and systematic hydration protocols (500 ml water intake), (2) complete caffeine and stimulant avoidance for 12 h preceding each session with parental coordination for compliance verification, (3) systematic coordination with academic schedules to minimize examination stress and academic pressure during testing periods, (4) standardized training protocols maintaining identical 48-hour recovery periods between sessions with coaching staff coordination, and (5) environmental consistency protocols ensuring identical court conditions, equipment, temperature, and lighting across all sessions.

Recognizing the inherent challenges of controlling confounding variables in adolescent elite athlete populations, additional measures included: parental coordination and education regarding protocol compliance, comprehensive participant education sessions regarding performance-affecting factors, flexible scheduling accommodating natural adolescent sleep patterns and academic commitments, systematic documentation of any routine variations or disruptions, and coaching staff collaboration to maintain training consistency. Despite these comprehensive control measures, perfect variable control remains challenging in real-world athletic contexts, representing an inherent limitation of ecologically valid research designs.

Our theoretical framework integrates established methodological approaches that remain foundational in contemporary research: circadian typology assessment using the extensively validated Horne-Östberg questionnaire (Horne & Ostberg, 1976, pp. 97–110), sleep pattern monitoring through proven diary methodology (Bastuji & Jouvet, 1985, pp. 299–305), and temporal mood variation research foundations (Hill & Hill, 1991, p. 434). The analytical framework employs distributed situational awareness theory (Salmon et al., 2017, pp. 23–45, 133–148), systematic qualitative analysis procedures (Strauss & Corbin, 1998, pp. 101–120, 158–175), and cours d’action methodology for examining situated cognition in dynamic environments (Theureau, 1992, pp. 45–78; Theureau, 2006, pp. 23–56). These methodological foundations provide validated frameworks essential for investigating collective cognitive coordination in team sports contexts.

### Data collection

Two types of data were collected for each match: audiovisual recordings, and verbalization data collected during the self-confrontation interviews that were subsequently conducted with each participant.

#### Audiovisual recording

The recording data was captured using two digital cameras. One was located on a fixed stand on the edge of the pitch near the opponent’s goal and was aimed at the attacking zone. The other was located in the middle of the pitch to provide a suitable shooting angle to track the players’ movements and record all their actions. This system enabled us to record all the actions and behaviors of our participants continuously.

#### Self-confrontation interviews

The self-confrontation interviews were based on a procedure in which each participant was subjected to an audiovisual recording of their activity. They are asked to describe and comment on their activity (Theureau, 1992; Theureau, 2006). The confrontation with the audiovisual traces favors the recall of the elements perceived during the game phases studied. In addition, these interviews last about 45 min after each match.

During the confrontation with the audiovisual recordings, each player is in an attitude and mental state that favors the explanation of his significant activity when he receives specific memories related to sensations, perceptions, focuses, concerns, emotions, and thoughts that accompany each action during the offensive phases. This type of questioning makes it possible to understand and describe the dynamics of the activity as it is experienced by the player at each moment.

### Data analysis

Offensive phase analysis focuses on field players engaged in coordinated attacking play, where cognitive coordination challenges are most evident and tactically significant. Goalkeeper’s cognitive demands during opponent attacks involve defensive positioning, anticipatory preparation, and individual decision-making rather than the collaborative cognitive coordination processes central to our investigation (Blecharz et al., 2022). Our audiovisual data collection system was optimized for capturing field player interactions and coordination patterns during attacking sequences. At the same time, goalkeeper activity occurred in different spatial zones with fundamentally different cognitive requirements. This focused approach maintains ecological validity for examining offensive cognitive coordination while enabling precise analysis of collaborative decision-making processes.

**Figure 1:**
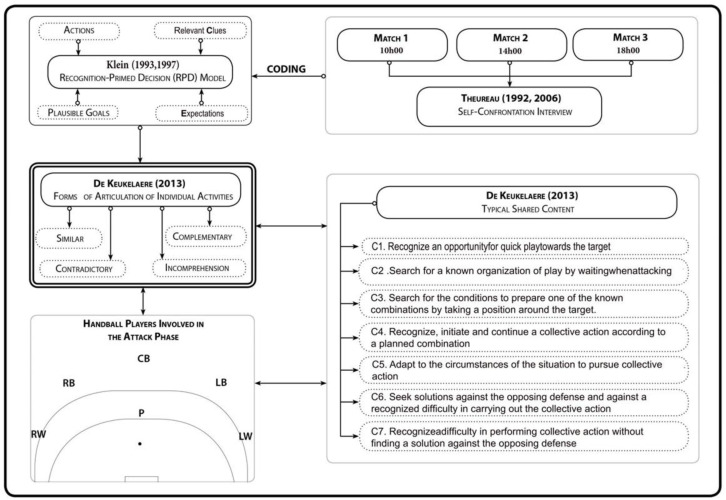
Experimental protocol design. Experimental protocol used in the study, detailing the steps and methodology for analyzing team coordination and decision-making in relation to diurnal variations.

The offensive phases of the three games were recorded and the sequences in which the six players were involved were analyzed. In this way, we were able to preserve ten sequences of the offensive phases in each match. The data were processed in the following steps: (1) elaboration of match chronicles, (2) coding of individual activities, (3) articulation of individual activities, (4) categorization of the data according to typical forms of sharing, (5) diachronic data analysis (Bourbousson et al., 2008; De Keukelaere et al., 2013) and (6) validation of the analysis (Fig. 1).

#### Elaboration of match chronicles

This step consists of preparing the data collected by verbalization in a table during each self-confrontation interview (Table 1).

The principle of reconstructing play chronicles is that for each action studied, there is a verbalization and an associated behavior. This step aims to make the data collected through verbalization and extrinsic observation usable. The placement of the players in this table shows the position of the players in the offensive phases.

#### Coding of individual activities

After reconstructing the match chronicles, the activity was “coded” by “cutting up” the verbalizations obtained into units of meaning that were considered significant from the player’s point of view. The coding was carried out according to the category system of the Recognition Primed Decision (RPD) model developed by Klein (1993) and Klein (1997), consisting of: typical actions (A), relevant clues (I), plausible goals (G), and expectations (EX).

**Table 1:** Reconstruction of match chronicle during Attack 2 at 10h00 (first half). Extract from the match chronicle reconstructed at 10h00, detailing player actions and interactions during the second attack phase. Ball circulation, positional shifts, and communication among players.

Match	Objective background	RW	RB	HC	P	LB	LW
ATTACK 2	The goalkeeper gets the ball and passes to RB. Ball circulation between players (RB-HC-LB). The two wingers move towards their wing to place themselves near the corner. HC announces the Yougo combination.	I’m going to stand on the side, the ball circulates in the center with LB, RB and HC.	I get the ball back, I pass to HC. Always at the same position, we circulate the ball slowly, to prepare the attack quietly.	I get the ball from RB. Wingers are not shifting yet. We knock the ball around to L and D.	Here, I’m taking place on the 6 m line, the ball is passed around between the two backs and HC, I stay close to my defenders. I request a pass from LB, but he doesn’t see me.	We pass the ball around; we have to move the defense.	When LB receives the ball, I move forward to give him a pass solution
	Departure of P from the 6–9 m zone. He crosses behind HC, receiving the ball, which he passes back to RB. P enters the defense. Crosses between HC and LB. RB passes to HC.	I’m watching the ball circulation. I’m focused on having the ball.	I pass the ball to HC. HC announces Yougo, so I have to get ready. P sends me the ball, I give it directly to HC.	I announce the Yougo combination with my hand, I cross with P and I pass him the ball. I have to change places with LB.	HC announces Yougo, so I have to get out of the defense zone and cross behind HC.	HC gives me a pass and I go to the HC position.	I stay on my wing and watch the game.

•  Typical actions correspond to verbs of actions. These units provide information about what the player does in a situation and what meaning he contributes to his action at a certain point in time t (*e.g.*, “I get the ball”). These units were coded with the letter A.

•  Relevant clues refer to the relevant contextual elements that the player identifies in the situation. This information refers to the opponents, the partners, or the player himself (*e.g.*, “I see the announcement of the combination”). These units were coded with the letter I.

•  Plausible goals refer to the player’s main concerns at that moment. In their formulations, these units were often preceded by the preposition “to”, thus emphasizing the intentional nature of the activity. Units expressing the player’s goal in a given situation were coded with the letter **G** (*e.g.*, “to destabilize the defenders”).

•  Expectations reflect potential events that are likely to occur in the immediate future. These expectations have a value of uncertainty or probability, and are often related to the activity of teammates or opponents. Units describing an expectation were coded with the letters EX (*e.g.*, “I’m waiting for a pass from the ARG”).

Qualitative coding employed the RPD model with systematic categorization: Actions (A)—specific behavioral verbs describing player activities and tactical behaviors, Relevant Cues (I)—contextual elements identified by players as situationally significant, Plausible Goals (G)—player intentions and tactical objectives at specific temporal moments, and Expectations (EX)—anticipated future events and teammate actions. Three researchers with extensive team sports analysis experience conducted coding following intensive training with the RPD model categorization and De Keukelaere et al. (2013) framework. Coding procedures included independent initial coding, systematic comparison of coding decisions, consensus meetings for discrepancy resolution, and comprehensive documentation of decision rationales.

Coding reliability was established through consensus procedures among three researchers for all categorization decisions. However, formal inter-coder reliability assessment (Cohen’s kappa coefficient) was not calculated, representing a significant methodological limitation for future enhancement. Target reliability standards (*κ* ≥ 0.80) should be implemented in future investigations to strengthen analytical validity. Despite this limitation, consensus procedures and systematic documentation provide reasonable confidence in coding consistency and analytical rigor for exploratory research purposes.

#### Articulation of the individual activities

Once the activities of the six players have been coded, this analysis consists of listing the actions of the handball players to identify moments of sharing and then comparing them based on the coding (I, A, G, EX) to determine whether the actions are related or not and whether the players’ goals and expectations converge or diverge. In this step, we can identify the shared contents and the modes of sharing by summarizing the elements of discourse shared by each player in a moment t (*e.g.*, the half-center shares an action with the left-back) based on the seven typical contents proposed by De Keukelaere et al. (2013). We found that these seven contents are divided into four forms according to this author. Our analytical focus on misunderstanding and divergence forms follows the validated theoretical framework of De Keukelaere et al. (2013), who categorized cognitive sharing into four forms: understanding and convergence forms (similar and complementary coordination) and misunderstanding and divergence forms (contradictory and misunderstanding coordination). Our research specifically investigated the latter category to examine performance breakdown mechanisms and identify opportunities for optimization. This focused approach enables a detailed analysis of coordination failures that significantly impact team effectiveness. Future investigations should study the complete spectrum of cognitive coordination forms.

Operational definitions: Contradictory form—players share identical contextual information and situational awareness but develop divergent interpretations and tactical responses, leading to uncoordinated actions despite shared initial perception and common situational understanding. Misunderstanding form—players assess situations using entirely different contextual information and situational cues, resulting in a complete absence of a shared tactical framework and fundamentally disconnected individual activities. Categories are mutually exclusive based on information sharing patterns and situational awareness overlap. Decision rules for ambiguous cases: the presence of shared initial information with divergent interpretation indicates a contradictory form, while the absence of information sharing with an independent situation assessment indicates a misunderstanding form. All classifications required a unanimous consensus among three researchers with comprehensive documentation of decision rationales.The seven contents categorized by the author are as follows:

• Recognize an opportunity to play quickly towards the target: These moments are based on the player’s understanding in the event of the opposing team losing the ball. The opponents did not have time to regroup in defense and the players participating in the study called an opportunity for a quick play towards the goal. These moments of sharing refer to quick counterattack situations where the team attempted to destabilize the opposing team quickly.
• Search for a known organization of play by waiting when attacking: these moments are characterized by the recovery of the ball after a stop by the goalkeeper or after a mistake by the opposing team. The players have then realized that the placement of a teammate or the quick retreat of the opposing team does not encourage them to launch a counter-attack. These moments of sharing imply a desire to keep the ball to engage the team in a placed attack.
• Look for the conditions to prepare one of the known combinations by taking a position around the target: These moments occur when players regain possession of the ball after a goal by the opposing team or when play resumes after a time-out. They are characterized by actions of ball circulation and placement depending on the player’s position. These moments of sharing refer to situations in which players wait for a spatial configuration about the goal to initiate a known combination.
• Recognize, initiate, and continue a collective action after a planned combination: these moments focus on the shared interpretations of the teammates after the announcement of a game plan or combination by the game maker (half-center). These moments of exchange make it possible to combine the anticipation of the actions with the execution of the combination.
• Adapt to the circumstances of the situation to act collectively: In these moments, the understanding of the individual situations is brought together, establishing the need to adapt to the circumstances. Often the combination did not go as planned. The contextual information considered relevant could refer either to the movement of a teammate or a space left by the opponent’s defense. These moments of sharing refer to the adaptation of several players to the context from a one-time mutual adjustment to allow advancement or access to the objective while facing the unpredictability of the opponents’ actions.
• Search for solutions against the opponent’s defense and against a perceived difficulty in carrying out the joint action: these moments describe the understanding of the individual situations in which the players explain the difficulties in carrying out the planned combination. These moments of exchange highlight situations in which the sequence of actions originally planned by the combination is not sufficient to destabilize the opponent’s defense and achieve the objective.
• Recognize a difficulty in performing a collective action without finding a solution against the opponent’s defense: These moments refer to interpretations of the players who were outmatched in executing the planned combination. The contextual information taken into account focuses on the difficulties encountered against the opponent’s defense. These moments of sharing are attributed to situations in which the originally intended game plan is no longer a sufficient resource to act and achieve the goal.
• Adapt to the circumstances of the situation to pursue collective action: These moments bring together understandings of individual situations stipulating the need to adapt to the circumstances. Oftentimes the combination did not go as planned. Contextual information, deemed relevant, could refer either to a movement of a teammate or a space left by the opposing defense. These moments of sharing refer to the adaptation of several players to the context from a one-off mutual adjustment to allow advancement or access to the target while facing the unpredictable nature of the actions of the opponents.
• Seek solutions against the opposing defense and against a recognized difficulty in carrying out the collective action: These moments describe the understandings of individual situations where the players explain the difficulties encountered in performing the planned combination. These moments of sharing highlight situations where the sequence of actions initially planned by the combination is not sufficient to destabilize the opposing defense and reach the target.
• Recognize a difficulty in performing a collective action without finding a solution against the opposing defense: These moments refer to interpretations made by the players who were defeated during the performance of the planned combination. The contextual information taken into account focuses on the difficulties encountered against the opposing defense. These moments of sharing are ascribed to situations where the game plan initially foreseen no longer constitutes a sufficient resource to act and reach the target.

Individual positional analysis revealed distinct patterns of cognitive coordination across player roles throughout different time periods. The half center (HC) and back players (RB, LB) demonstrated higher involvement in contradictory forms during morning sessions, reflecting their central roles in tactical coordination and information processing. Wing players (RW, LW) showed more consistent coordination patterns across time periods, potentially due to their more specialized positional responsibilities. The pivot (P) exhibited unique coordination challenges during morning sessions, particularly in spatial positioning and timing expectations with back players.

Table 1 presents detailed individual positional contributions to cognitive coordination dynamics, demonstrating how each playing role (RW, RB, HC, P, LB, LW) contributes to collective offensive coordination and highlighting position-specific patterns in contradictory and misunderstanding forms across different temporal conditions. This analysis reveals that coordination difficulties are not uniformly distributed across positions but reflect specific tactical responsibilities and information processing demands inherent to each playing role.

#### Categorization of data according to typical forms of sharing

This analysis consists of preparing in a table the different shared moments of each offensive phase to deduce the typical forms of sharing, which are the object of this study, namely (Table 2):

**Table 2:** Characteristics and elements of the RPD model in typical forms of sharing. Forms of articulation of individual activities in team play, focusing on contradictory and misunderstanding forms. Characteristics, elements of the RPD model under review, and the influence of shared or unshared contextual information on coordination and expectations.

Forms of articulation of individual activities	Characteristics	Elements of the RPD model under review
Contradictory	Characterized by: • Sharing of contextual information. • The divergence of interpretations. *i.e., players shared contextual information but interpreted differently.*	Divergent goals (G) which are raised by the player. • Expectations (EX) not being met.
Misunderstanding	Characterized by: • No sharing of contextual information. • The divergence of interpretations. *i.e., players interpreted the situation from different contextual information.*	• The action (A) of a partner does not present relevant information (I) for the player. • The absence of shared (I) generates divergent expectations (EX) and uncoordinated actions (A). Expectations (EX) not being met.

#### Diachronic data analysis

This step allowed a dynamic description of the evolution of the shared content and the typical forms of sharing as the situation progressed (the different moments of a handball match). This analysis is performed using a temporal graph representing the different contents and typical forms of sharing as well as the sharing modes between the members of the team.

#### Validity of the analysis

The validity of the analysis is based on the study by De Keukelaere et al. (2013), which was confirmed and verified by the reliability of the categorization of meaning units (Strauss & Corbin, 1998) and by the triangulation procedure between researchers familiar with the study and the team sport.

This study employed an explanatory sequential mixed-methods design systematically integrating quantitative frequency analysis of cognitive coordination patterns with qualitative mechanistic exploration through self-confrontation interviews. Data integration was achieved through methodological triangulation across three distinct sources: external observation (systematic audiovisual behavioral analysis), participant introspection (structured self-confrontation verbalization), and researcher interpretation (systematic coding validation using the RPD framework). Integration procedures included correlating quantitative pattern frequencies with qualitative thematic analysis, verifying consistency between observed coordination difficulties and participant-reported cognitive experiences, and systematically identifying convergent and divergent findings across data sources to ensure a comprehensive understanding.

Triangulation validation employed systematic procedures: (1) quantitative coordination pattern frequencies correlated with qualitative verbalization themes to identify mechanistic explanations, (2) temporal sequence analysis comparing observed behavioral coordination with participant-reported cognitive processes, (3) cross-validation of coordination difficulties through multiple analytical perspectives, and (4) researcher consensus procedures involving three investigators familiar with team sports methodology to ensure analytical rigor. This triangulation approach strengthens validity through convergent evidence across multiple data sources and analytical frameworks.

### Statistical analyses

Chi-square (*χ*2) was used to examine how the decision-making patterns identified by the RPD model differed in the morning, midday, and evening. In particular, we examined how misunderstandings and disagreements differed in the team’s interactions at these different times of the day.

## Results

### Variability of the elements of the recognition-primed decision model according to the “contradictory and misunderstanding” forms

In regards to the actions, our results suggest differences between these three moments of the day (*χ*2(G) = 93.5; *p* < 0.001) and that contradictory and misunderstanding forms are more common at 10h00 (67%) than at 14h00 (21%) and 18h00 (12%).

Concerning the relevant clues, findings also suggest the difference between these three moments of the day (*χ*2(G) = 139.93; *p* < 0.001) again with the contradictory and misunderstanding forms being more common at 10h00 (67%) than at 14h00 (21%) and 18h00 (12%).

Again, a similar pattern emerged for plausible goals, with differences between the three moments of the day (*χ*2(G) = 53.95; *p* < 0.001). Likewise, the contradictory and misunderstanding forms are more common at 10h00 (71%) than at 14h00 (19%) and 18h00 (10%).

As with the previous results, there were differences across the three moments of the day in terms of expectations (*χ*2(G) = 70.05; *p* < 0.001) and contradictory and misunderstanding forms are more common at 10h00 (79%) than at 14h00 (12%) and 18h00 (9%).

**Figure 2:**
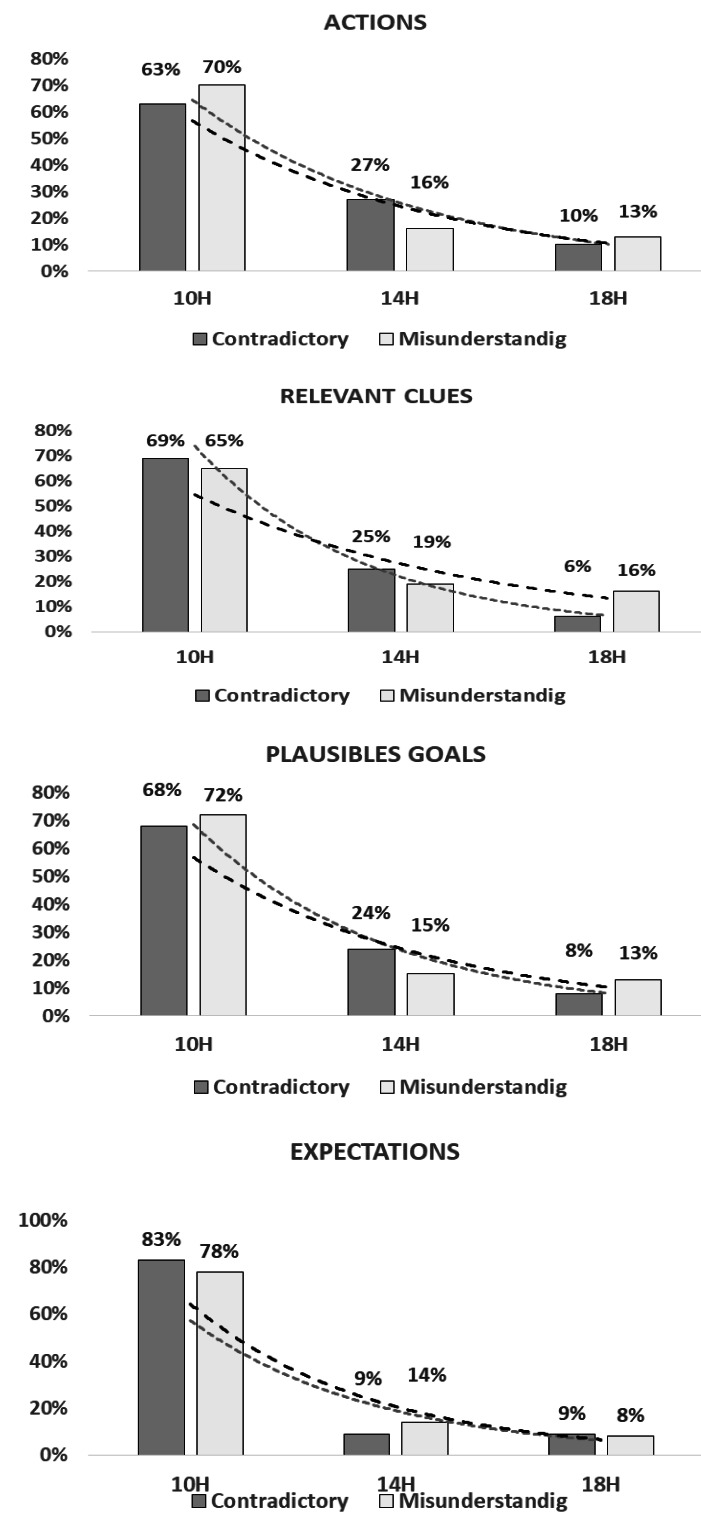
Diurnal variation in RPD model elements. Diurnal variation of the elements within the RPD model, categorized by the contradictory and misunderstanding forms of coordination, demonstrating how these forms evolve across different times of the day.

Given our intensive case study design (*n* = 6), traditional inferential statistics are inappropriate due to low statistical power and assumption violations. We present descriptive statistics with effect size estimation, providing preliminary evidence for future confirmatory studies: Morning sessions (10h00) demonstrated higher frequencies with large effect sizes: contradictory forms 67% (10/15 instances, Cohen’s *h* = 0.89), misunderstanding forms 71% (15/21 cases, Cohen’s *h* = 0.95). Afternoon sessions (14h00): contradictory 21% (3/15), misunderstanding 19% (4/21). Evening sessions (18h00): contradictory 12% (2/15), misunderstanding 11% (2/21). Large effect sizes indicate practically meaningful differences warranting replication studies with appropriate statistical power for definitive conclusions (Fig. 2).

### Sharing modes

The analysis of the articulation of individual activities allowed for the identification of 44 local forms of articulation in the morning, 27 forms in the afternoon, and 31 in the evening. These different forms were classified according to the articulation of typical forms of situation awareness developed by De Keukelaere et al. (2013).

**Figure 3:**
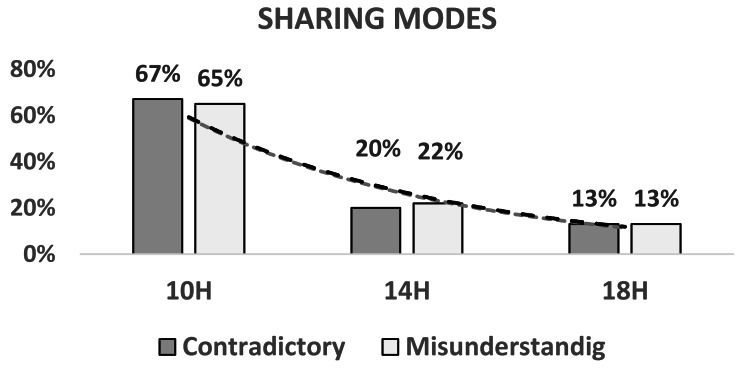
Temporal evolution of sharing modes in contradictory and misunderstanding forms. Progression and variability of sharing modes within the contradictory and misunderstanding forms throughout the day, emphasizing changes in coordination and contextual information sharing.

The computed chi-square suggested differences between the three different times of the day (*χ*2(G) = 18.37; *p* < 0.05) and there were more contradictory and misunderstanding forms at 10h00 (66%) than at 14h00 (21%) and 18h00 (13%) (Fig. 3, Table 3).

**Table 3:** Time-of-day analysis of variability in contradictory form of coordination. Main elements of the contradictory form of activity articulation, focusing on the sharing of contextual information and divergence of interpretations at three time points (10h00, 14h00, and 18h00). Analysis of the evolution of sharing levels throughout the day.

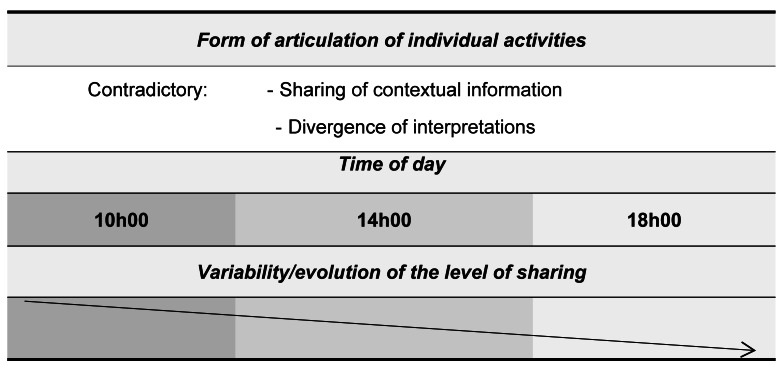		

### The contradictory form

In this form, characterized by a sharing of contextual information and a divergence of interpretations, the players mobilized divergent goals and unfulfilled expectations.

According to the results, inter-individual differences (De Keukelaere et al., 2013) in the construction of knowledge during action were noted (Sève et al., 2002; Bourbousson & Sève, 2010). These differences have a high frequency at 10h00 compared to 18h00. At these moments, players share the same contextual information but do not interpret it in the same way. “I try to pass the ball back to half-center (G), I find myself blocked by two defenders (I), so I pass to pivot (A)” (Right back); “…I never waited for a pass from right back (I). I’m not available (I)…he gives me a low pass (I)” (Pivot) (Half-time 1, 3rd offensive phase, 10h00); “I am waiting for pivot to intervene (EX) to support me (G)…” (Left back), “…I think left back finishes the action on his own (EX)” (Pivot) (Half-time 2, 7th offensive phase, 18h00).

In the morning, the players shared six cognitive elements and were frequently engaged in a collective activity of two to three players based on “seeking solutions against the opposing defense and facing a difficulty in carrying out collective action”, “recognizing a difficulty in carrying out collective action without finding a solution against the opposing defense” and “seek conditions to prepare one of the known combinations by taking a position around the target”. In the afternoon, during the second offensive phase, the players were engaged in the context in which the pivot goes towards the offensive zone and crosses behind the half-center who passes. But the pivot loses the ball. So, two game priorities were recorded: “recognize, trigger, and continue a collective action according to a planned combination” and “adapt to the circumstances of the situation to continue the collective action (from the situation initiated)”. It is important to note that during the evening, these players shared in the form of contradictory articulation a single moment “recognize a difficulty in the realization of the collective action without finding a solution against the opposing defense” (Table 4).

**Table 4:** Time-of-day analysis of variability in misunderstanding form of coordination. Key elements of the misunderstanding form of activity articulation, emphasizing the lack of contextual information sharing and divergence in interpretations. The variability in sharing levels is analyzed across three time points (10h00, 14h00, and 18h00).

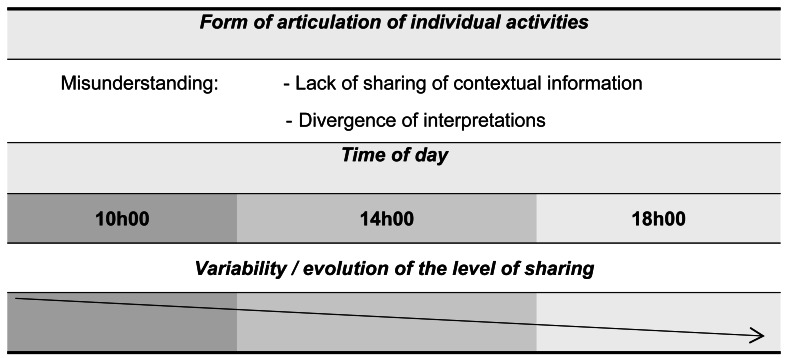		

### The misunderstanding form

For this form of articulation, and following the results obtained, a divergence in the coordination of the individual activities of the players was observed. This divergence was found to be significant and high at 10h00 compared to the other two moments of the day, *i,e.*, at 14h00 and 18h00.

It is important to point out an absence of common links (Sève et al., 2009) between the members of a team. These results reinforce the idea of the deprivation of an understanding of the situation, highlighting the involvement of the players in most of the typical content of sharing based on the successive imbalances in the organization of the game “…I’m waiting for left back to send me the ball (EX)…Left back can’t see me at all (I)” (left winger); “…I have to shoot on my own (G)” (left back); “I’m a little fed up in attack and I don’t know what to do…” (half-center) (Half-time 1, 4th offensive phase, 10h00).

Such misunderstandings mean that relevant information and knowledge are not shared. It is characterized by moments of dysfunction at the level of the player’s collective activity (Bourbousson et al., 2011). According to the results, Poizat et al. (2008) proposed a form of non-sharing in co-authorship with a table tennis player, but this non-sharing was defined by a lack of information manifestation and reduced trust.

The collective activity at 10h00 needs two to four players. It is characterized by uncertainty related to the actions of the players and then a notable lack of effectiveness “…I want him to pass me back the ball (EX), I see that left back is playing without any coordination …(I)” (half-center); “…I am preparing to cross the block of defense (G)…I would like to pass to pivot (EX)” (left back); “…I’m getting ready to have a pass (EX), left back can’t see me (I), I’m alone (I)…” (Left winger) (Half-time 2, 10th offensive phase, 10h00). The teammates did not collectively refer to the plan, and disconnected from a common frame of reference (De Keukelaere et al., 2013).

## Discussion

This study aimed to investigate the effects of the timing of cognitive content, in particular the forms “contradictory” and “misunderstanding”, on the performance of elite male handball players at different times of the day (10h00, 14h00 and 18h00). These times correspond to the usual competition schedules of the federations (Essid et al., 2022). In choosing the forms of cognitive sharing, we rely on De Keukelaere et al. (2013), who categorized them into four forms grouped as: understanding and convergence forms (*i.e.,* similar and complementary coordination) and misunderstanding and divergence forms forms (*i.e.,* contradictory and misunderstanding coordination).

Our analysis showed that cognitive sharing and understanding in elite male handball players depended on the time of day. Afternoon sessions were characterized by improved cognitive sharing and understanding. This improvement contrasts with the morning sessions, which were more often associated with forms of cognitive sharing that were either inconsistent or indicative of misunderstanding. This finding is consistent with diurnal patterns previously reported for cognitive and physical performance, mood, and vigor, which are better in the afternoon in young elite male handball players (Essid et al., 2022). Such diurnal variations in knowledge-sharing and decision-making highlight the significant influence of cognitive and perceptual elements on team dynamics.

The forms of cognitive sharing, “contradictory” and ”misunderstanding”, were predominantly observed in the morning, suggesting a link between the timing of cognitive sharing and the natural circadian rhythms that influence physical and mental performance (De Keukelaere et al., 2013). A higher incidence of different interpretations and coordination challenges among players characterized games played in the morning. This pattern suggests that the time of day has a direct impact on team synergy and performance, with the morning hours presenting particular difficulties for effective team coordination and understanding.

Recent investigations in chronopsychology have demonstrated significant diurnal variations in cognitive performance variables, with peak cognitive functioning occurring during specific circadian phases corresponding to individual chronotype preferences (Munnilari et al., 2024). These findings align directly with our observations of improved cognitive coordination during afternoon and evening sessions compared to morning periods. Contemporary research further demonstrates that circadian rhythms significantly influence executive function, working memory capacity, and attention control, with optimal performance typically occurring during late afternoon and early evening hours (Schmidt et al., 2021; Facer-Childs et al., 2021).

Furthermore, the application of the RPD model, which focuses on typical actions, relevant clues, plausible goals, and expectations, provided a nuanced perspective on the sharing of knowledge and content during the offensive phase of matches (Klein, 1993; Klein, 1997). The frequent observation of misunderstanding and divergence forms forms of cognitive sharing in the morning session highlights the potential to align team activities with athletes’ optimal cognitive functioning times to improve performance.

Contemporary team cognition research emphasizes shared mental models, collective information processing, and cognitive coordination as fundamental determinants of team performance outcomes (Filho & Tenenbaum, 2020). Recent investigations demonstrate that successful teams exhibit superior collective cognitive capabilities (Blecharz et al., 2022; Trecroci et al., 2021), including enhanced communication, coordinated attention, and synchronized decision-making processes (Gray, Helper & Osborn, 2020; Rico-González et al., 2021). These theoretical developments support our investigation of cognitive coordination patterns as indicators of collective performance capacity and provide framework for understanding how circadian factors influence team cognitive functioning.

The current understanding of the influence of circadian rhythms on athletic performance indicates that most physiological and cognitive parameters peak during the evening hours, significantly affecting team interaction and coordination processes (Ayala et al., 2021). Recent research specifically examining team sports contexts reveals that circadian synchronization among team members enhances collective performance outcomes, supporting our investigation of temporal factors in cognitive coordination (Vitale et al., 2022; Vogel & Schack, 2023).

The contradictory form was particularly pronounced in the morning sessions, highlighting a clear influence of the time of day on team dynamics and cognitive processes. The “contradictory” form was characterized by players working towards different goals and dealing with unmet expectations. Our findings echoed previous research indicating inter-individual differences in the construction and sharing of knowledge during gameplay (Sève et al., 2002; Bourbousson & Sève, 2010; De Keukelaere et al., 2013). These differences were most evident in the morning (10h00), a time when players share the same contextual information but often do not interpret it consistently. For example, a right wing-back attempted to play a pass into the middle but was blocked and opted to pass to the pivot, who in turn was not expecting the pass and was therefore not in an optimal position to receive it.

A left wing-back waiting for an intervention from the pivot, which did not happen, was also an example of the different expectations and actions of team members. In the morning sessions, players were often confronted with collective activities that were fraught with misunderstandings and unaligned objectives, such as searching for solutions against the opponent’s defense without finding effective solutions or preparing known combinations without correct execution. In contrast, in the afternoon sessions, players showed a more coherent approach, especially in adapting to situational changes that required deviating from the planned course of action.

This variation in cognitive sharing, with a higher incidence of “contradictory” forms in the morning, suggests a link to natural circadian rhythms that influence physical and mental performance. The lower vigor scores and increased negative mood states observed in the morning (Chtourou et al., 2018; Essid et al., 2022) support the idea that these early hours present additional challenges for effective team coordination and cognitive alignment.

Our analysis revealed that the time-of-day influences players’ physical and mental readiness of players and significantly affects the nature of cognitive sharing within the team settings. The morning hours, which are associated with lower energy levels and a more negative mood, appear to exacerbate the challenges of achieving cognitive synchrony between team members. In contrast, the afternoon and evening sessions, which align more closely with the peak performance of these athletes, allow for a more harmonious and effective form of team interaction and decision-making. The form of misunderstanding showed a significant gap in team coordination that was particularly pronounced at 10h00, as opposed to later times. This pattern replicates the finding of Sève et al. (2009), who suggest a deficit in shared connections and mutual understanding, which are critical for teamwork and effective performance. The data suggest a notable mismatch in the team’s synchronization efforts during morning sessions, resulting in suboptimal execution of game strategies. Instances, where players’ expectations of each other’s moves were not met, highlighted the difficulties in promoting unified team actions, leading to frustration and reduced efficiency. These incidents highlighted a failure to adequately share important information and align on common goals, which manifested as operational dysfunction within team activities, as described by Bourbousson et al. (2011). For instance; Poizat et al. (2008) similarly identified a form of “non-sharing” in team sports, characterized by an apparent lack of information sharing and diminished trust.

The requirement for a coordinated effort between two to four players during 10:00 a.m. sessions was characterized by widespread uncertainty about players’ actions, leading to significant inefficiency. Examples of this included a player stating that they needed a pass that never came, or attempting to position themselves for a strategic move that was not supported by teammates, representing a disconnect from the planned collective approach (De Keukelaere et al., 2013). This scenario illustrates the lack of a common strategic framework, which makes effective team collaboration and performance.

The constant interactivity between the players gave rise to a certain degree of uncertainty, which tended to increase. In this context, the differences observed at the level of the two contradictory and misunderstanding forms were explained by the results of previous studies that had shown a correlation between the time of day and cognitive performance (Barthe, Quéinnec & Verdier, 2004; Kline et al., 2010), characterized by better reaction time scores in the afternoon (*i.e.,* 14:00 and 20:00). In the same context, Essid et al. (2022) used Stroop and reaction time tests to show that young handball players perform better in the afternoon and evening than in the morning. Another explanation for the high deviations in the morning is consistent with previous studies that have investigated diurnal differences in mood states (Hill & Hill, 1991; Chtourou et al., 2014; Chtourou et al., 2018). They showed that in sedentary subjects, the aspect of negative moods (*i.e.,* tension, depression, anger, fatigue, and confusion) and overall disturbance of mood were higher in the morning than in the afternoon.

In the morning, the players were no longer able to maintain a high level of performance due to various factors, such as fatigue. In this context, Gawron, French & Funke (2001) have shown that fatigue in the central and peripheral forms can lead to a decline in physical performance in the task studied. Similarly, Meeusen et al. (2006) claimed that central fatigue was associated with a decrease in performance on tasks involving cognitive functions.

In this study, the concept of collective cognition in this handball team was equated with experimenting at three different times of the day. This approach aimed to determine the time at which the highest level of collective understanding was reached, indicating optimal collective performance.

We propose a three-level mechanistic cascade linking circadian rhythms to team cognitive coordination: (1) individual level—circadian fluctuations in executive function (working memory, attention control, cognitive flexibility) create temporal windows of optimal information processing capacity varying across morning, afternoon, and evening periods; (2) dyadic level—individual cognitive variations create temporal misalignment in information sharing, interpretation accuracy, and coordination timing between player pairs engaging in tactical interactions; (3) team level—accumulating dyadic misalignments manifest as increased contradictory and misunderstanding coordination patterns during morning hours when individual cognitive resources are suboptimal, while afternoon/evening periods facilitate synchronized cognitive functioning enabling enhanced collective coordination performance.

Alternative explanations for observed coordination patterns include simple fatigue accumulation effects, learning confounds from repeated testing exposure, motivational variations related to daily scheduling preferences, environmental factors (such as temperature and lighting variations), and social dynamics that are independent of biological circadian rhythms. However, consistency with established chronobiology research on individual cognitive performance, alignment with team cognition theory regarding collective information processing requirements, and correspondence with our previous psychometric findings (Essid et al., 2022) support the interpretation of a circadian mechanism, while acknowledging the multicausal complexity in real-world performance contexts.

The results of this study demonstrate the influence of time of day on team members’ shared understanding, depending on the two forms of articulation of each activity. We found two predominant forms in the morning at 10h00 compared to the afternoon at 14h00 and the evening at 18h00. These were the contradictory and misunderstanding forms, which were due to divergences in the understanding of the situation and the coordination between the players.

These findings highlight a critical aspect of collective performance, particularly when considering the primary risk factors for sleep deprivation in athletes, allowing for more targeted interventions (Reis et al., 2021; Walsh et al., 2021). Athletes’ sleep is affected by sport-specific factors such as training, travel, and competition, as well as non-sport-specific factors such as gender, stress, and anxiety. This consensus leads to a toolkit for practitioners that includes sleep education and screening to mitigate these risk factors and optimize athletes’ sleep. In our study, this is critical for optimal and efficient sharing of cognitive content for collective performance, especially as participants were asked to maintain their sleep patterns with at least seven hours of sleep during the experimental period, maintain their habitual physical activity, and avoid any strenuous activity the day before each testing session.

## Limitations

Although our investigation of diurnal variations in cognitive sharing among elite male handball players provides novel insights, it is important to recognize its limitations. The specific focus of the study on six elite male field players was strategically chosen to reflect the core of a handball team, as the dynamics of cognitive sharing unfold mainly among these players. This selection aims to capture the complex team dynamics and cognitive processes that are critical for handball performance.

Despite comprehensive control measures, achieving perfect control over all potential confounding variables was not feasible, given the complex nature of adolescent daily routines and real-world athletic contexts. Our intensive case study methodology (*n* = 6) has profound implications for establishing reliability and statistical generalizability, which require careful interpretation within appropriate analytical frameworks. A fundamental methodological limitation concerns distinguishing between genuine anticipatory expectations and *post-hoc* rationalizations generated during self-confrontation interviews, despite the use of audiovisual anchoring procedures. The absence of objective sleep monitoring, while addressed through comprehensive self-report protocols, represents a significant constraint on definitive circadian mechanism attribution. Future investigations should incorporate objective physiological monitoring, larger samples based on appropriate power analyses, comprehensive control for confounding variables, and enhanced integration of mixed-methods to address current methodological limitations while building upon these exploratory findings.

However, this approach limits the scope of our findings to this specific setting and excludes the role of the goalkeeper, which could provide additional insights into overall team coordination and performance.

Our intensive case study design (*n* = 6) represents an established approach in elite athlete research, enabling a comprehensive analysis of cognitive coordination patterns that would be impossible with larger samples due to the methodological intensity requirements. While limiting statistical generalizability, this methodology provides unprecedented analytical depth and generates valuable hypotheses for future confirmatory research. The observed large effect sizes (Cohen’s *h* = 0.89−0.95) indicate practically meaningful differences warranting replication studies with appropriate statistical power (target *n* ≥ 30 per time condition), positioning our findings as hypothesis-generating rather than definitive conclusions.

The absence of objective sleep and activity monitoring (actigraphy, polysomnography, sleep diaries) represents a significant methodological limitation that constrains our ability to attribute coordination patterns to circadian mechanisms definitively. Control measures included detailed sleep hygiene instructions (a minimum of 7.5 h of sleep duration, consistent bedtimes), pre-session verbal compliance verification, coordination with parents and coaching staff to ensure protocol adherence, and systematic documentation of any reported sleep disruptions. However, relying on self-reported compliance introduces potential bias and uncontrolled variations that could confound the effects of time of day. Future investigations should incorporate objective sleep monitoring technology to ensure rigorous assessment of the circadian rhythm.

Our intensive case study methodology has important implications for establishing reliability and stability, which require careful interpretation. The small sample size limits statistical generalizability and increases the susceptibility to individual participant variations that can affect overall patterns. However, the rich qualitative data from extensive self-confrontation interviews provide detailed mechanistic insights into cognitive coordination processes that larger quantitative studies cannot achieve. These findings should be interpreted as preliminary evidence that establishes theoretical foundations and generates hypotheses for future confirmatory research with appropriately powered samples.

The self-confrontation methodology has inherent limitations, including potential retrospective bias, where participants may rationalize or reconstruct cognitive processes rather than accurately recall in-the-moment cognition. Additional concerns include social desirability bias and individual differences in introspective ability that may systematically affect data quality and validity. However, our approach minimizes these limitations by conducting immediate post-match interviews with audiovisual anchoring, which reduces memory decay and reconstruction effects compared to delayed recall methods. Validation through methodological triangulation, comparing verbalized cognitive processes with observed behavioral coordination patterns, provides additional confidence in data authenticity. However, complete elimination of retrospective bias remains impossible in any methodology requiring verbal cognitive reports.

The self-confrontation interview technique specifically addresses anticipatory cognition *versus post-hoc* rationalization through immediate audiovisual re-immersion, anchoring participant recall to specific perceptual cues and temporal contexts. Structured questioning protocols focus on contemporaneous concerns and situational awareness rather than outcome evaluation, minimizing retrospective reconstruction. Validation involves temporal sequence analysis, comparing stated expectations with subsequent behavioral patterns, and triangulation with observed coordination behaviors. However, complete elimination of rationalization risk remains impossible in any methodology requiring verbal cognitive reports, representing an inherent limitation requiring acknowledgment in result interpretation.

In addition, the study focused on cognitive misunderstandings and inconsistencies and omitted other cognitive aspects such as creativity and problem-solving, which are also critical to team success. Future research should include these dimensions for a more comprehensive understanding of team dynamics.

External factors such as training schedules, academic commitments, and psychological states that could influence cognitive performance and team dynamics were not considered. Future research should incorporate these variables to develop a more holistic model of team performance in sports.

While this study builds upon the comprehensive psychometric validation from our previous research with the same participants, concurrent physiological monitoring represents an essential enhancement for future investigations. Direct physiological measures, such as heart rate variability, cortisol sampling, continuous core body temperature monitoring, and electroencephalographic assessment, would strengthen documentation of the circadian rhythm and provide mechanistic insights into the observed cognitive coordination patterns. Future studies should integrate real-time physiological markers with cognitive coordination assessments to establish more robust circadian influence documentation.

## Conclusion

Examining the effects of time of day on cognitive sharing among elite male handball players in this study provides valuable information for improving team performance. Our results suggest that cognitive misunderstandings and inconsistencies occur less frequently in the afternoon and evening, indicating that these times are optimal for team activities that require high levels of coordination and understanding.

Sports coaches and practitioners can capitalize on these findings by scheduling tactical training and critical decision-making exercises at these optimal times to improve team effectiveness.

##  Supplemental Information

10.7717/peerj.20370/supp-1 Supplemental Information 1 Raw data examining the influence of diurnal variations on cognitive coordination and misunderstandings among elite male handball players Performance metrics, cognitive decision-making assessments, and observational data recorded at different times of the day (10:00, 14:00, and 18:00). The dataset is structured to capture variations in team dynamics, reaction times, decision-making accuracy, and communication efficiency. Each entry represents an individual player’s recorded data during controlled match simulations, with anonymized identifiers ensuring confidentiality.

10.7717/peerj.20370/supp-2 Supplemental Information 2 Verbalization of the two forms of misunderstanding and contradiction in team sports Detailed verbalization of the two forms of misunderstanding and contradiction in team sports interactions. It includes sequences of play, contextual objectives, and player responses, categorized based on their verbal expressions during gameplay. Highlights instances where coordination breaks down, leading to errors in execution, and provides insights into the cognitive processing of players in real-time situations.

10.7717/peerj.20370/supp-3 Supplemental Information 3 Verbalization coding of misunderstanding and contradictory forms in decision-making during handball match sequences Detailed verbalization analyses from player interactions during handball match sequences across three time points (10:00, 14:00, and 18:00). Includes the coding of player utterances based on four components of the Recognition-Primed Decision (RPD) model: Actions (A), Relevant Clues (I), Plausible Goals (G), and Expectations (EX). It identifies instances of misunderstanding and contradictory forms of shared cognition, the degree of coordination, and the sharing modes. Figures 2 and 3 provide quantitative summaries of the frequency and temporal variation of these elements.

10.7717/peerj.20370/supp-4 Supplemental Information 4 Coding and categorization of misunderstanding and contradiction in team play scenarios Verbalized interactions in team play scenarios, focusing on misunderstanding and contradiction as key factors influencing game dynamics. It includes coded sequences of actions, player involvement, and shared or divergent expectations. Systematically organizes the identified forms of misunderstanding and contradiction, contributing to the analysis of decision-making processes in collective sports.

## Additional Information and Declarations

### Data Availability

The following information was supplied regarding data availability: The raw measurements are available in the Supplemental Files.

## References

[ref-1] Araújo D, Davids K, Renshaw I (2020). Cognition, emotion and action in sport: an ecological dynamics perspective. Handbook of sport psychology.

[ref-2] Ayala V, Martínez-Bebia M, Latorre JA, Gimenez-Blasi N, Jimenez-Casquet MJ, Conde-Pipo J, Bach-Faig A, Mariscal-Arcas M (2021). Influence of circadian rhythms on sports performance. Chronobiology International.

[ref-3] Barthe B, Quéinnec Y, Verdier F (2004). L’analyse de l’activité de travail en postes de nuit: bilan de 25 ans de recherches et perspectives. Le Travail Humain.

[ref-4] Bastuji H, Jouvet M (1985). Interet de l’agenda de sommeil pour l’etude des troubles de la vigilance. Electroencephalography and Clinical Neurophysiology.

[ref-5] Blecharz J, Wrześniewski K, Siekańska M, Ambroży T, Spieszny M (2022). Cognitive factors in elite handball: do players’ positions determine their cognitive processes?. Journal of Human Kinetics.

[ref-6] Bossard C, De Keukelaere C, Cormier J, Pasco D, Kermarrec G (2010). L’activité décisionnelle en phase de contre-attaque en Hockey-sur-glace. Activités.

[ref-7] Bourbousson J, Feigean M, Seiler R (2019). Team cognition in sport: how current insights into how teamwork is achieved in naturalistic settings can lead to simulation studies. Frontiers in Psychology.

[ref-8] Bourbousson J, Poizat G, Saury J, Sève C (2008). Caractérisation des modes de coordination interpersonnelle au sein d’une équipe de basket-ball. Activités.

[ref-9] Bourbousson J, Poizat G, Saury J, Sève C (2011). Cognition collective: partage de préoccupations entre les joueurs d’une équipe de basket-ball au cours d’un match. Le Travail Humain.

[ref-10] Bourbousson J, Sève C (2010). Construction/déconstruction du référentiel commun d’une équipe de basket-ball au cours d’un match. EJRIEPS.

[ref-11] Chtourou H, Aloui A, Hammouda O, Souissi N, Chaouachi A (2014). Diurnal variation in long- and short-duration exercise performance and mood states in boys. Sport Sciences for Health.

[ref-12] Chtourou H, Engel FA, Fakhfakh H, Hammouda O, Ammar A, Sperlich B (2018). Diurnal variation of short-term repetitive maximal performance and psychological variables in elite judo athletes. Frontiers in Physiology.

[ref-13] Cooke NJ, Gorman JC (2006). Assessment of team cognition. International Encyclopedia of Ergonomics and Human Factors.

[ref-14] Cooke NJ, Gorman JC, Myers CW, Duran JL (2013). Interactive team cognition. Cognitive Science.

[ref-15] De Keukelaere C, Kermarrec G, Bossard C, Pasco D, Loor P (2013). Formes, contenus et évolution du partage au sein d’une équipe de sport de haut niveau. Le Travail Humain.

[ref-16] Dergaa I, Fessi MS, Chaabane M, Souissi N, Hammouda O (2019). The effects of lunar cycle on the diurnal variations of short-term maximal performance, mood state, and perceived exertion. Chronobiology International.

[ref-17] Dergaa I, Varma A, Musa S, Chaabane M, Salem AB, Fessi MS (2020). Diurnal variation: does it affect short-term maximal performance and biological parameters in police officers?. International Journal of Sport Studies for Health.

[ref-18] Essid S, Cherif M, Chtourou H, Souissi N (2022). Time-of-day effects in physical performances and psychological responses in young elite male handball players. Biological Rhythm Research.

[ref-19] Facer-Childs ER, De Campos BM, Middleton B, Skene DJ, Bagshaw AP (2021). Temporal organisation of the brain’s intrinsic motor network: the relationship with circadian phenotype and motor performance. NeuroImage.

[ref-20] Filho E, Tenenbaum G (2020). Team mental models: theory, empirical evidence, and applied implications. Handbook of sport psychology.

[ref-21] Gawron V, French J, Funke D (2001). Stress, workload, and fatigue. An overview of fatigue.

[ref-22] Gray R, Cooke NJ, McNeese NJ, McNabb J (2017). Investigating team coordination in baseball using a novel joint decision-making paradigm. Frontiers in Psychology.

[ref-23] Gray JV, Helper S, Osborn B (2020). Value first, cost later: total value contribution as a new approach to sourcing decisions. Journal of Operations Management.

[ref-24] Hill CM, Hill DW (1991). Influence of time of day on responses to the profile of mood states. Perceptual and Motor Skills.

[ref-25] Horne JA, Ostberg O (1976). A self-assessment questionnaire to determine morningness-eveningness in human circadian rhythms. International Journal of Chronobiology.

[ref-26] Klein GA (1993). A recognition-primed decision (RPD) model of rapid decision making. Decision making in action: models and methods.

[ref-27] Klein G (1997). The recognition-primed decision (RPD) model: looking back, looking forward. Naturalistic decision making.

[ref-28] Klein G (2008). Naturalistic decision making. Human Factors.

[ref-29] Kline CE, Durstine JL, Davis JM, Moore TA, Devlin TM, Youngstedt SD (2010). Circadian rhythms of psychomotor vigilance, mood, and sleepiness in the ultra-short sleep/wake protocol. Chronobiology International.

[ref-30] Meeusen R, Watson P, Hasegawa H, Roelands B, Piacentini MF (2006). Central fatigue. Sports Medicine.

[ref-31] Mhenni T, Michalsik LB, Mejri MA, Yousfi N, Chaouachi A, Souissi N, Chamari K (2017). Morning–evening difference of team-handball-related short-term maximal physical performances in female team handball players. Journal of Sports Sciences.

[ref-32] Munnilari M, Bommasamudram T, Easow J, Tod D, Varamenti E, Edwards BJ, Ravindrakumar A, Gallagher C, Pullinger SA (2024). Diurnal variation in variables related to cognitive performance: a systematic review. Sleep and Breathing.

[ref-33] Poizat G, Sève C, Serres G, Saury J (2008). Analyse du partage d’informations contextuelles dans deux formes d’interaction sportives: coopérative et concurrentielle. Le Travail Humain.

[ref-34] Portaluppi F, Smolensky MH, Touitou Y (2010). Ethics and methods for biological rhythm research on animals and human beings. Chronobiology International.

[ref-35] Przednowek K, Śliz M, Lenik J, Dziadek B, Cieszkowski S, Lenik P, Kopec D, Wardak K, Przednowek KH (2019). Psychomotor abilities of professional handball players. International Journal of Environmental Research and Public Health.

[ref-36] Reis M, Ramiro L, Paiva T, Gaspar-de Matos M (2021). National survey on the importance of sleep in the quality of academic life and mental health of college students in Portugal. Sleep Science.

[ref-37] Rico-González M, Ortega JP, Nakamura FY, Moura FA, Los Arcos A (2021). Identification, computational examination, critical assessment and future considerations of spatial tactical variables to assess the use of space in team sports by positional data: a systematic review. Journal of Human Kinetics.

[ref-38] Roenneberg T, Wirz-Justice A, Merrow M (2003). Life between clocks: daily temporal patterns of human chronotypes. Journal of Biological Rhythms.

[ref-39] Romdhani M, Dergaa I, Moussa-Chamari I, Souissi N, Chaabouni Y, Mahdouani K, Abene O, Driss T, Chamari K, Hammouda O (2021). The effect of post-lunch napping on mood, reaction time, and antioxidant defense during repeated sprint exercise. Biology of Sport.

[ref-40] Salas E, DiazGranados D, Klein C, Burke CS, Stagl KC, Goodwin GF, Halpin SM (2008). Does team training improve team performance? A meta-analysis. Human Factors.

[ref-41] Salmon PM, Stanton NA, Walker GH, Jenkins DP (2017). Distributed situation awareness, theory, measurement and application to teamwork, volume 23–45.

[ref-42] Scharfen HE, Memmert D (2019). Measurement of cognitive functions in experts and elite athletes: a meta-analytic review. Applied Cognitive Psychology.

[ref-43] Schmidt MH, Dekkers MP, Baillieul S, Jendoubi J, Wulf MA, Wenz E, Fregolente L, Vorster A, Gnarra O, Bassetti CL (2021). Measuring sleep, wakefulness, and circadian functions in neurologic disorders. Sleep Medicine Clinics.

[ref-44] Sève C, Bourbousson J, Poizat G, Saury J (2009). Cognition et performance collectives en sport. Intellectica—la Revue de L’Association Pour la Recherche sur Les Sciences de la Cognition.

[ref-45] Sève C, Saury J, Theureau J, Durand M (2002). La construction de connaissances chez des sportifs de haut niveau lors d’une interaction compétitive. Le Travail Humain.

[ref-46] Souissi A, Dergaa I, Musa S, Saad HB, Souissi N (2022). Effects of daytime ingestion of melatonin on heart rate response during prolonged exercise. Movement & Sport Sciences-Science & Motricité.

[ref-47] Starkes JL, Ericsson KA (2003). Expert performance in sports: advances in research on sport expertise.

[ref-48] Strauss A, Corbin J (1998). Basics of qualitative research: techniques and procedures for developing grounded theory.

[ref-49] Tenenbaum G, Basevitch I, Gutierrez O (2015). Cognitive capabilities. Encyclopedia of sport and exercise psychology.

[ref-50] Theureau J (1992). Le cours d’action, analyse sémio-logique: essai d’une anthropologie cognitive située. Lang Bern.

[ref-51] Theureau J (2006). Le cours d’action: Méthode développée.

[ref-52] Trecroci A, Duca M, Cavaggioni L, Rossi A, Scurati R, Longo S, Merati G, Alberti G, Formenti D (2021). Relationship between cognitive functions and sport-specific physical performance in youth volleyball players. Brain Sciences.

[ref-53] Vitale JA, Galbiati A, De Giacomi G, Tornese D, Levendowski D, Ferini-Strambi L, Banfi G (2022). Sleep architecture in response to a late evening competition in team-sport athletes. International Journal of Sports Physiology and Performance.

[ref-54] Vogel L, Schack T (2023). Cognitive representations of handball tactic actions in athletes—the function of expertise and age. PLOS ONE.

[ref-55] Walsh NP, Halson SL, Sargent C, Roach GD, Nédélec M, Gupta L, Samuels CH (2021). Sleep and the athlete: narrative review and 2021 expert consensus recommendations. British Journal of Sports Medicine.

