# Supplementary material for: Influence of diurnal variations on cognitive coordination and misunderstanding in elite male handball players
Source: PeerJ. 2026 Jan 15;14:e20370. doi: 10.7717/peerj.20370 (PMC12812274; doi:10.7717/peerj.20370)
Supplement: Supplemental Information 2 — Detailed verbalization of the two forms of misunderstanding and contradiction in team sports interactions. It includes sequences of play, contextual objectives, and player responses, categorized based on their verbal expressions during gameplay. Highlights instances where coordination breaks down, leading to errors in execution, and provides insights into the cognitive processing of players in real-time situations. [file peerj-14-20370-s002.docx]

| **MATCH 1** | | | | | | | | | |
| --- | --- | --- | --- | --- | --- | --- | --- | --- | --- |
|  |  |  |  |  |  |  |  |  |  |
| **M1** | **Sequences** | **Context Objective** | **RW** | **RB** | **HC** | **P** | **LB** | **LW** |  |
| **ATTACK 1** | A1.2 | HC is located between two defenders from the opposing. HC made a dribble, and switches back to RB. RB begins to dribble away to the right and goes to HC, which backs up to the median line. | Here, the opposing defense is very high **(I)**, I still have on my wing **(EX)** | HC makes me a pass **(I)** , I find myself blocked by four defenders**(I)**. I don't know what to do **(I)** because RW is marked by an opponent **(I).**  And then, I see HC back to the midline **(I)** to help me **(G)**, it is free **(I)**, I give him the ball **(I)** | Je make a dribble **(A)** and I advance toward the area of 6-9m **(A)**, I want to give the ball to P **(EX),** I find myself stuck **(I)**, you play as two back **(I)**, I try to pass to LW **(EX),** and I seeTheG is far from the action **(I)**, I turn on the D **(A)** to look for another partner **(G)**, I advance **(A),** then I make a pass at RB **(A)**.  I see RB is not free **(I)**, it is surrounded by four defenders **(I)**, je back to the midline **(A)** I try to provide a solution to pass**(EX)** | As I'm in between two defenders **(I)**, I concentrate to keep a good position **(G)**, I pay attention to guys but also to DC **(EX)** | I took two minutes I am not involved in this attack,  I look at the game off the field | I look at what is happening **(Has),** I see DC is blocked **(I)**, I go out of my wing **(A), I** trying to help him **(EX)** then I move **(A)** for him to offer a solution **(G).**  HC don't see me **(I),** he gives the ball to RB **(I).** |  |
|  | | | | | | | | | |
| **M1** | **Sequences** | **Context Objective** | **RW** | **RB** | **HC** | **P** | **LB** | **LW** |  |
| **ATTACK 2** | A2.3 | HC made a dribble away to the right, it is marked by a defender. HC passes the ball to RB, who sends it directly to RW. RW shifts to the line of 9m and a high pass HC. | RB sends me to the ball **(I)**, I is marked by an opponent **(I)**, I move **(A),** I am headed to the line of 9m **(A)**, to share the ball with HC **(G)** , and then I give him a high-pass **(A)** | HC deviates on the right **(I)**, it aims RW in its visual field **(I)** and suddenly I see that I excluded from this action **(I).**  I get a pass unexpectedly HC **(I)**, I go straight to RW **(A)**  I step back three steps **(A)** pending the happening of RW **(EX)** but unfortunately RW is beyond me **(I)** | I have the ball **(I)**, I digress to the right **(A)**, I want to give the ball to RW **(EX)** but I find myself blocked by two defenders **(I)**, all of a sudden, RB is near me **(I),** I advance **(A)** and I can't find another solution **(I)** than to make a pass at him **(A)** | I am stuck between two defenders **(I)**. J’trying’to be available for my partner, **(G)** | Je look at the game carefully **(A)** | I am running the game **(A)** and I remain attentive **(EX)**, I could have a ball **(EX)** |  |
|  |  |  |  |  |  |  |  |  |  |
| **M1** | **Sequences** | **Context Objective** | **RW** | **RB** | **HC** | **P** | **LB** | **LW** |  |
| **ATTACK 3** | A3.3 | LB receives the ball, he found before him a defender, he sends the ball to RB. RB is a dribble, and is blocked by two defenders. RB made a low pass in P who finds himself surrounded by three defenders. P is shoved in the back by an opponent. The coach blows the whistle. | The RB pass was not successful **(I)**, it is destroyed by an opponent **(I)**. Fault **(I)** | HC sends me to the ball **(I)**, I make a feint to my direct opponent **(A)**, I digress on the left **(A)**, I try to pass the ball to HC **(G)**, I find myself blocked by two defenders **(I)**, so I'm making a pass to P **(A)**  I think we found a quick solution **(EX)**, But unfortunately, it was not successful **(I)**, we lose the ball **(I)**. | I start running towards the area of 6-9m **(A)** to go to the shooting **(G)**, I find myself blocked by a defender **(I)**, I make a pass at RB **(A)**.  I'm waiting for a pass from the RB **(EX),** I put my hands in front **(A)** to provide a pass solution **(G)**, as a result, RB, reacting without thinking **(I)**.  We lose the ball **(I).**  We start the work with my partners in cooperation and then each one of us wants to show himself | As usual, I position myself on the defence area **(A)**, marked by two defenders **(I)**, waiting for an opportunity to exchange with my partners **(EX)**.  I am the movement of the ball by making attention to the rear **(A)** that could send me to the ball **(EX)**.  As I find myself surrounded by three defenders **(I),** I never expected a pass from RB **(I)**. I'm not available **(I)**.  All of a sudden, I made a low pass **(I).** Simply, RB does not know what to do | I am prepared to receive the ball **(EX)**.  I’am waiting a pass from RB **(EX),** I will lift my hands **(A)** to attract his attention, **(G)** and provide a pass solution **(G),** but to no avail**.** I'm a little tired of it. | I follow the movement of the ball **(A)** |  |
|  |  |  |  |  |  |  |  |  |  |
|  |  |  |  |  |  |  |  |  |  |
| **M1** | **Sequences** | **Context Objective** | **RW** | **RB** | **HC** | **P** | **LB** | **LW** |  |
| **ATTACK 4** | A4.1 | RW recovers the ball and race to the line of 9m. RW pass the ball to RB ahead and deviates to the left and a high pass to LB. | I get the ball **(A)** and I try to go faster **(A)** to realize the rise of fast ball **(G)**, the decline of the opposing defense is done quickly **(I)**, as a result, RB is a bit off **(I)**, I stalling speed **(A)** in order to seek a partner **(G)**, I step back to make a pass at RB **(G)**.  I gives the ball to RB **(A)**, I start running towards my wing **(A)** , and I expect that I redid a pass **(EX)** | RW part in the race **(I),** I would like to leave quickly **(EX)** for it to support **(G).**  RW is advanced compared to me **(I)**, as a result, I expect it to go to the tire **(EX)**, even when I try I accelerate to receive a ball **(G)**  RW makes me a pass **(I)**, I make a dribble **(A)**, I digress to the left **(A)**, I find myself blocked by two defenders **(I)**,I see LB free **(I)**, I give him a pass **(A)** | I would like to run quickly **(EX)** to receive a ball **(G)**  I wait for RB makes me a pass **(EX)**, I diverge to the right **(A)**, I put my hands in front **(A)**, RB crosses his race in front of me , without any regard **(I).** | I'm in the race **(A)** to position myself on the line 6m **(G)** | I find myself on the left **(I)**  I prepare myself to receive the ball **(EX)**, my arms are in front **(I)**, I expect a pass from RB **(EX)** | I'm in the corner **(A)** to stretch a maximum of defence **(G)** |  |
|  | A4.2 | LB made a feint to pass his direct opponent. He jumps and pulls the line-of-9m. No goal. | I’m far from the action **(I)**, I stay on my wing **(EX)**  Oh, we missed the chance **(I)** | I wait for LB, to pass me the ball again **(EX)**, I put the hands in the air **(A).**  LB plays alone **(I)** and pulls **(I)**  The action of pulling of LB is unexpected | I've got a little bit tired in attack and I don't know what to do.  Je rest on my place **(EX)** | Here, I have nothing to do, **(I)**  I expected that this action takes place otherwise | I get the ball RB **(I)**, I go in the race moving forward in the line of 6m **(A)**.  Ofthem defenders are in front of me **(I)**, RB calls me **(I)**, I must be going in the tire only **(G)**, I jump **(A)** and I take **(A)**, not goal **(I)** | I put on my wing **(A)**, I expect that LB sends me the ball **(EX)** since I had enough space on the line 6m **(I)**  LB don't see me at all **(I)** |  |
|  |  |  |  |  |  |  |  |  |  |
| **M1** | **Sequences** | **Context Objective** | **RW** | **RB** | **HC** | **P** | **LB** | **LW** |  |
| **ATTACK 5** | A5.1 | RB recovers the ball and passes it to HC. HC advance towards the area of 6-9m. He is stopped by two defenders. The coach throws the arm. | Like every time, I'll be in my corner **(A)**, to stretch a maximum of defence **(G)** | I see HC is blocked by a defender **(I)**, I expect that it will make me a pass **(EX),** I lift up my left hand **(A)** to have a ball **(G)**, but HC back is turned **(I)** and headed to the left **(I)**. | I get the ball from RB **(I)**, I advance toward the line of 9m **(A)**  I find a defender in front of me **(I)**, I try to exceed, to go to the tire **(G)**, it is preventing me from moving forward **(I).**  The coach blows the whistle **(I)** | I am in the defence zone between the two opponents **(I)**.  I'm waiting for a pass from HC **(EX)**  I try to be available to HC **(G)** in order to provide a solution that password **(G)**, but he doesn't see me **(I)**.  The coach blows the whistle **(I)**. | I’am prepared to receive the ball **(EX),** my arms are in front **(I)**. I see that HC is looking to surpass his opponent **(I).** Then, I step back **(A)**. | I put on my wing **(A)** |  |
|  | A5.2 | P recovers the ball and passes to HC that the returns directly to LB. LB starts to dribble by pushing to the line of 6m. He is blocked by two defenders, made a low pass in P. P is shoved in the back by a defender. He falls. The coach blows the whistle on the misconduct. There is a penalty. | I stay in my corner **(EX)** | I remain on the right **(EX)**, I look at the game **(A)** | I am preparing to announce a combination **(G)** but LB upsets all the work **(I)**.  I crossed my race with LB **(A)**, I place myself on the left **(A).**  I'm waiting for a pass from LB **(EX)**, I must prepare myself to help him **(G).**  LB continues to play all alone **(I)**.  The ball is lost **(I)** | I get the ball **(A)** and pass to HC **(A)** to re-start the game **(G)**. Then, I go back on the line 6m **(A)**  I rest on my position **(EX)**  In Spite of that I find myself blocked by three defenders **(I)**, LB made a low pass **(I)**, I try to recover **(G)** but I have not been able to **(I)**. An opponent pushes me **(I)**, the ball is lost **(I)** | I meet with HC **(A).** He gives me the ball **(I)**, I tries to give it back **(G)**.  Since it is a little decreased **(I)**, I am preparing to advance into the line of 6m **(G).**  I am blocked by three defenders **(I)**, I make a feint to exceed **(A)** and then I'm making a low pass to P **(A).** | I look at the game **(A)** |  |
|  |  |  |  |  |  |  |  |  |  |
| **M1** | **Sequences** | **Context Objective** | **RW** | **RB** | **HC** | **P** | **LB** | **LW** |  |
| **ATTACK 6** | A6.2 | P fate of the defence. RB is a dribble and sends P. P is blocked by two opponents. It is a high pass to RB. The ball drops. RB recovers the ball and passes it to RW | I put myself on the right wing **(A)** and I look forward to a future balloon **(EX)** | I have the ball **(I)**, I try to make a pass to HC **(G)**, I see a defender who is close to him **(I)** and as a result, PVT comes out of the box 6-9m to support me **(I)**, and I give him a pass **(A)**  P advance to the line of 6m **(I)**, when he takes the position of jump shooting **(I)**, I expect it to go to the tire **(EX)**. P gives me a pass unexpected **(I)**.  The ball drops **(I)**, I get it **(A)** and I will pass directly to RW **(A)** | Here, I find myself far from the action **(I)**, I remain ever watchful waiting for a pass **(EX)**  Je finds thate the start of P has no meaning. | I see RB surrounded by two defenders **(I)**, donc I'm leaving the defence area **(A)** to provide a solution that password **(G).**  I get the ball **(I**), I find before me three defenders **(I)**, so I gives him the ball **(A)** | I lift up my hand **(A)** in order to attract the attention of RB **(G)** and that it will make me a pass **(G)**. The defenders are distant **(I)**, I would like to go to the tire **(EX)**, no one sees me **(I)** | I am near the corner **(I)**, I look at the placement of game **(A)** |  |
|  | A6.4 | HC makes a dribble and goes to P which part of the defense zone. P crosses behind HC and sends the ball to RB, and then returned to the line of 6m. RB begins to dribble by pushing to the line-of-9m, he finds a space and pull in. No goal. | Imust that I stay on my wing **(EX)**, because I could have a pass **(G)** | I get the ball P **(I)**, I try to give back to HC **(G).** I'm ready to go to the tire **(EX)**, I make a feint **(A)** to switch my direct opponent **(G)** and I shoot from the line of 9m **(I)**.  A'i, ai, ai... I missed my chance **(I)** | There, I am announcing Yougo **(A)**, LB passes me the ball **(I)**, I dribble **(A),** I expect **(EX)** as P arrives and crosses behind me **(I),** I gives him the ball **(A)**.  I meet with LB **(A)**  I expect a pass from RB **(AT)**, so I prepare to go in the race **(G)** and exchange the ball with him **(G)**.  I see that’ RB plays only **(I)** | Where, HC announces the combination Yougo **(I)**, I have to leave right away **(G)**, HC makes me a pass **(I)**, I crossed behind him **(A)**, I give the ball directly at RB **(A)**, then I go on the defence area **(A)** | I see the announcement of Yougo **(I)**, I must prepare myself to make the crossover with HC **(G)**  I raise my hands **(A)** I request the ball **(A)**, no one sees me **(I)**, I'm fed up in attack  I have always the same problem with the RB, it is in the diceagreement. | LB is found only on the left **(I)**, here, I think that RB passes LB **(** **EX)**  Oh the the..., everyone wants to show |  |
|  |  |  |  |  |  |  |  |  |  |
| **M1** | **Sequence** | **Context Objective** | **RW** | **RB** | **HC** | **P** | **LB** | **LW** |  |
| **ATTACK 7** | A7.2 | LB is a dribble, moving forward in the right and goes to RB. RB is a dribble, moving forward in the area of 6-9m, is blocked by two opponents, he throws the ball. | I advance on the side towards RB **(A)**, I am preparing to have a pass **(EX)**, because there is a space in front of me **(I)** to go shoot **(G)** | I retrieve the ball of ARG **(I)**.  I am preparing to go to the tire **(G)**, so I'm moving forward **(A).** I find myself blocked by three opponents **(I)**, so I throw the ball on the left **(A)**. Fortunately, LB get **(I)** | LB sends me to the ball **(I)**, I try to recover **(EX)**, so I have a screen delay **(I)** and I miss the ball **(I)**  Here, I expect a pass from RB **(EX),** so I'm preparing for the receive **(EX),** of a sudden, he throws the ball without any reflection**. (I).** It amazes me.  I can't understand my partners back (LB, RB) | I find myself between two opponents who are glued to me **(I)** | I’m moving towards the area of 6-9m **(A)**, I find before me a defender who is preventing me from getting to the line 6m **(I)**, so I have to make a pass to HC **(G)**  In this action, there was a misunderstanding with HC, it was intended to play Yougo **(I)**, we are going to repeat again the action | There, I look at what is happening **(A)**  LB begins to play quickly without stalling the game **(I)** |  |
|  | A7.3 | LB recovers the ball and began to dribble. He makes a pass at LW by crossing which it reverts back to HC. Movement of ball between HC-LB-RB.  HC announces Yougo. | I am not involved in this action **(I)**, so I'm trying to watch the game **(A)** | I raise my hands to the top **(A)** to attract the attention of LB **(G)**, but LB don't see me **(I)**. He decides to play alone **(I)**.  I'm running the ball with HC and LB in the area of 9m **(A)** | I am preparing for a pass from LB for the second time **(EX)**, I have the arm in front **(I)**, LB made me a feint of pass **(I)**, I feel that it is blocked **(I).** LB goes to LW **(I)**.  Finally**,** LW makes me a pass **(I)**, we run the ball **(A)** before that I announce **(A)** what we must play in attack **(I)** | I tried tobe available for my partner, **(G)** | I get the ball **(A)**, I’m doing a dribble **(A)**, LW is near me **(I)**, I give him a pass (**A).**  LW does not understand what are we going to play now, I would like to if it remains on its wing quietly **(EX),** it's going to cause us discomfort at the level of movement | When LB is receiving the ball **(I)**, I am ready to receive a pass **(EX)**, I’m moving toward LB **(A)**, it gives me the ball **(I)**, I crossed behind him **(A)** and I may give it directly to HC **(A)**, then I go back on my wing **(A)** |  |
|  | A7.5 | HC made a feint to pass his opponent and goes to RB in crossing each other. RB deviates to the left and made a pass to LB. LB move towards to the line of 6m, it is blocked by his opponent, he made a feint to pass the opponent and pulls. No goal. | I put on my wing **(A)** and I look at how the game is played **(A)** | I receive the ball from HC **(I)**, I diverges on the left **(A)**, I'm doing a dribble by pushing to LB **(A)**, I give him a pass **(A)** | RB gives me the ball **(I)**, I make a dribble **(A)**, I digress to the right **(A)**, I gives him the ball **(A)** taking its place **(I)** | I see RB has the ball **(I)**, I lift up my hand **(A)** for give me a pass **(G)** so that I could go to the tire **(G)**.  RB passes LB **(I)**. For the second time **(I)**, I leve my two hands, **(A)** in order to attract the attention of RB **(G)**, RB continues to play all alone **(I)**.  RB rate of mark **(I)** | Mes arms are in front **(I)**, I expect a pass from RB **(EX)**.  RB sends me to the ball **(I)**. Je finds a defender in front of me blocking me **(I)**, I managed to overcome it by a passage of arms **(A)**, in order to go to the tire **(G)** , I find myself near the line of 6m **(I)**, I jump **(A)** and I take **(A)**. ...Uffff... I miss my opportunity **(I)**. | I always stay on my wing **(EX)**, I expect a pass **(EX)**.  When RB is receiving the ball **(I)**, I'm preparing right away to have a password LB **(EX)**. |  |
|  |  |  |  |  |  |  |  |  |  |
| **M1** | **Sequences** | **Context Objective** | **RW** | **RB** | **HC** | **P** | **LB** | **LW** |  |
| **ATTACK 8** | A8.1 | LB recovers the ball and passes it to LW which is a dribble, and give directly, then in his corner to corner. LB makes a feint to pass his opponents, and then begins to dribble by pushing to the line of 6m. He is stopped by two defenders. The coach blows the whistle to continue the game. | Here, the game it doesn't work well between my partners | Where’LB advance to the line of 6m **(I)**, I lift up my hand **(A)** so that it sends me the ball **(G)**, because I find myself in a good position **(I)** to go to the tire **(G)**, LB not see me **(I)** | I see that LB has the ball **(I)** and advance to the defence zone **(I)**, I go in the race at the same time **(A)**, I expect it will give me a pass **(EX)** to move forward **(G)** and go to the tire **(G)**, it is a failure by the defendant **(I)**. Fault (**I)**. | I try always to be available for the one who has the ball **(G)**, I remain attentive **(EX)** to propose solutions at the right time for my partner, **(G)** | I get the ball from LW**(I)**, I advance towards the area of 6-9m **(A)**, I’m doing a dribble **(A)**, I am looking for a position **(A)** to penetrate between the defenders on the line of 6m **(G)**, I find myself blocked by two opponents **(I**). I tries to make a pass at HC**(EX)** but I can't **(A).**  Here, I'm waiting for HC deviates on the left **(EX)** but it stays in the center **(I)**, the ball is lost **(I).** | Before I place myself on the side **(I)**, LB passes me the ball **(I)**, I may give **(A)**, I get back on my wing **(A)** |  |
|  |  |  |  |  |  |  |  |  |  |
| **M1** | **Sequences** | **Context Objective** | **RW** | **RB** | **HC** | **P** | **LB** | **LW** |  |
| **ATTACK 9** | A9.1 | LB recovers the ball and runs quickly toward the area of 6-9m. Feint of LB to pass his direct opponent, he is blocked by two opponents. LB makes a high pass to HC. HC made a feint and doing a dribble, and then sends the ball to RB. | LB plays alone **(I).**  Here, ithere is no sharing between my partner, this is not done, we are a team | I look at what is there **(A)** but I am shocked of what takes place in attack | I see lb plays very quickly without sharing **(I)** as usual. I'm waiting for a pass of his hand **(EX).** But, it plays only **(I)**, it really astonished me  LB is blocked **(I)**, finally, it makes me a pass **(I)**, I find before me four opponents **(I)**, I make a feint **(A)** to pass the opponents **(G)**, I turn to right **(A)**, I pass the ball to RB **(A)** | I keep a good position **(I)** in order tobe available for LB **(G)** I plan to have a pass to the LB **(EX)** and give it as a solution of passes **(G)**  LB continues to play alone **(I)** | I get the ball **(A)**, I try to find a solution **(G),** I foresee that I could go to pull **(EX)**, I began to dribble forward **(A)** to the line of 6m **(I)**, there are three opponents that I block **(I)**, I try to move forward **(EX)** and then I do a high pass to HC **(A).**  Here, HC remains on its position **(I)** instead of spreading on the left.  I am waiting for the crossover **(EX)**, | I ask a thousand and one times to give me the ball **(A)**, no one sees me **(I)** |  |
|  | A9.2 | RB made a gesture with his hands to stall the game. Movement of ball between RB-HC-LB-HC-LB-RB | I place on my wing quietly **(A)** | I receive the ball from HC **(I)**, I make a gesture with my hand **(A)** to delay the game **(G)**, I go to HC **(A)**  I get the ball to the LB **(I)**, I advance towards the centre **(A)**, I found a defender in front of me **(I)**, I make a pass at HC **(A)** , which is a little spread on the left **(I)** | RB passes me the ball **(I)**, I may give directly to LB **(A)**  We pass the ball to the left and the right **(A)**  RB gives me the ball **(I)**, I argue **(A)** and I am planning ofgoing to the tire **(G)**, I find myself on the line 6m **(I)**, I draw **(A)** | As I am marked by two opponents **(I)**, I try to put myself in position **(G)** to be available to my partner **(G)** | I get the ball of HC **(I)**, I may give directly **(A)**, HC gives me the ball again **(I).**  Here, I would like to make a pass to LW **(EX)**, but as I find myself placed in the wrong position **(I)**, so I make a high pass to RB **(A)**. | I put myself on the wing G **(A)** , waiting for an opportunity to pass **(EX)**  The defenders are distant **(I)**, there is an opportunity of draws **(I)**, I call LB a lot of time **(A)** to have a pass **(G),** and provide a solution **(G)**, but unfortunately, his position did not allow him to make me a pass **(I)** |  |
|  | A9.3 | RB makes a pass to HC. HC advance to the line of 6m and deviates to the left. He is blocked by two opponents and pulls. No goal. | I rest in my corner **(EX)** and I find myself far from the action **(I)** | HC sends me the ball **(I)**, I wish to make a pass to P **(EX)** which is located on the line of 6m **(I)** and as I am blocked by two defenders **(I)**, I send the ball to HC **(A)**  I expect that HC gives me back the ball **(EX)** | RB sends me to the ball **(I)**, I hopes to go to the tire only **(EX)**, I advance **(A)** to the area of 6-9m **(I)**, I try to go to the fire **(EX),** then I make a feint **(A)** to switch my direct opponent **(G)** and I pull **(A)**.  Unfortunately, I missed the goal **(I)** | I put the arm in air **(A)** in order to attract the attention of my partner, **(G)** | I am prepared to receive a ball of HC **(EX)**, I advance a step **(A)**, and then I see HC advance to the line of 6m quickly **(I)**, he prepares for the tire **(I)** | I stay on my wing **(EX)**, I look at my partner **(A)** |  |
|  |  |  |  |  |  |  |  |  |  |
|  |  |  |  |  |  |  |  |  |  |
| **M1** | **Sequences** | **Context Objective** | **RW** | **RB** | **HC** | **P** | **LB** | **LW** |  |
| **ATTACK 10** | A10.2 | LB hand in the race to the zone 6-9m, is blocked by two defenders. He passes the ball P that is blocked by an opponent. P is shoved in the back. The coach blows the whistle. | I'm far enough away from the action **(I)**, I stay on my wing **(EX)** | I look at what is going on with LB **(A)** | I go to LB **(A)**, I want him to pass the ball back to me **(EX)**, I see that LB plays without any coordination with me **(I)**, the same problem always with him, I rest in the center without moving **(EX)** | LB sends me to the ball **(I)** despite the fact that I'm not available **(I)**, I try to retrieve the ball **(G)**, the defender pushes me in the back **(I)**, the ball falls off **(I)**, it is a mistake **(I)** | HC gives me the ball **(I)**, in that moment, I am preparing to cross the block defence **(G)**, I find myself blocked by two defenders **(I)**, there is no space **(I)** to go shoot **(G)**, I would like to make a pass to P **(EX)** | When I see LB advance with the ball **(I)**, I prepare myself to have a pass **(EX)**  LB not see me **(I)**  I am alone **(I)**, there is space for away from here **(I)** |  |
|  |  |  |  |  |  |  |  |  |  |
|  |  |  |  |  |  |  |  |  |  |
| **MATCH 2** | | | | | | | | | |
|  |  |  |  |  |  |  |  |  |  |
| **M2** | **Sequences** | **Context Objective** | **RW** | **RB** | **HC** | **P** | **LB** | **LW** |  |
| **ATTACK 2** | A2.1 | On the median line, P makes a pass at HC. All players move towards the line of 9m. HC makes a pass to RB. RB pass again to RW, which is placed on the right wing and passes it on directly. RB passes the ball to HC. Movement of the ball between players RB-HC-LB-LW.  HC announces the combination Yougo. | RB passes me the ball **(I)**, I brought back directly **(A)**, and then I place myself in my corner **(A)** | When it returns to the ground **(I)**, we run the ball to the left and to the right **(A)** | P recovers the ball **(I)** and makes me a pass **(I)**, I start running towards the front **(A)**, I go to RB **(A)** that the returns to RW **(I)**, and then he gives me back the ball **(I)**, is made to circulate the ball **(A)** and I am preparing to announce a combination **(I).**  Here, I am announcing Yougo **(A)** with my hand almost three times **(I)**, but P don’t see me **(I)**, his gaze is focused on a something else **(I),** I try to find a solution **(EX)** | I go quickly to the defence zone **(A)** to place me near my defenders **(B)** and I keep my position **(I).**  I’m waiting for the announcement of HC **(EX)** | We pass the ball **(A)** as usual  P does not follow HC | I put on my wing **(A)**.  Je get the ball **(I)** and I may give directly to LB **(A)** |  |
|  | A2.2 | P hand to the attack area and crosses his race behind the HC, which makes him a pass. P loses the ball. | I position myself on my wing **(A)**  Ufff... the ball drops **(I)** | I look at the game of HC and P **(A)**    When the ball drops **(I)**, I have to go in the race **(G)** to recover the ball **(G)**, after I see LB recovers the ball **(I)** | I am announcing again the Yougo combination **(A)** with my hand and my voice **(I)** to attract the attention of P **(G)**  in order to facilitate the exchange of the ball **(G).** Je see P moves **(I).**  I am preparing **(A)** to throw the ball to P **(G)** , and then to cross the race with LB **(G)** | I see the announcement **(I)** and I hear the word ‘soon’ from HC **(I)**, and then he raises his hand **(I).**  I’m off to the race **(A)** to the area of attack **(I)**, I crossed behind HC **(A)**, I expect the ball **(EX)**, I have a screen delay **(I)** and kick the ball drops **(I)**.  I does not happen to catch up with my hands **(A)**,I have tried **(A),** I step back towards the median line **(A)** to recover **(G)**, a defender pushes me in the back **(I).** I fell in **(A).** | I am preparing to cross my race with HC **(G)**, I see that the ball drops **(I)**, I go in the race **(A)** to retrieve it **(G)** | I'm with attention my partner **(A)** |  |
|  | A2.3 | HC recovers the ball on the halfway line, makes a dribble, and sends it to LB. LB is found in the advanced position compared to HC. The ball is lost. | There, I encourage my partners **(A)** | I am far from the action **(I)**, I look at what is happening **(A)** | I get the ball **(A)**, I begin to dribble **(A)** I advance **(A)** to the line of 6m **(I)**, and hop I make a pass to LB **(A**) , which keeps a good position **(I)** to go fast **(G)**, I don't know what he has, he can't catch up the ball **(I)** | Oh, no, this is not possible, one loses the ball **(I)** | I see that HC has the ball **(I)**, I must prepare myself **(A)** to provide a solution that password **(G)**, I'm running **(A)** to attract his attention, **(G)**, it throws me the ball too behind **(I)**, I try to catch up **(A),** of the coup, I have a screen delay **(I)**, I missed the ball **(I)** | Oh there, we lose the ball for a second time **(I)** |  |
|  |  |  |  |  |  |  |  |  |  |
|  |  |  |  |  |  |  |  |  |  |
| **M2** | **Sequences** | **Context Objective** | **RW** | **RB** | **HC** | **P** | **LB** | **LW** |  |
| **ATTACK 3** | A3.3 | RB makes a pass to HC which is advancing rapidly and made a feint to pass his direct opponent and pulls. No goal. | This is not a goal **(I)** | When HC advance **(I)**, I crossed behind him **(A)**, I expect it will give me a pass **(EX**) to go to the tire **(G)**  I would have liked to finish the attack to the outside | I get the ball **(I)** and I’m advancing rapidly **(A)**, I doing a dribble **(A).** Hop, there's a defender in front of me **(I)**, my goal is for him to exceed **(G)**, so I'm making a feint **(A)**. I remain cautious **(I).**  I advance into the line of 6m **(I)** and I pull **(A)**.  Unfortunately, I completely missed my pulls **(I)** | I try to keep a good position between the defenders **(G)** to be always available for my partner **(G).**  Oh no, HC misses to score **(I)**, no goal **(I)** | Phew, HC can't score the goal **(I)**, another chance **(I)** | HC has managed to exceed the power of defence **(I)**  It misses the tire **(I)**, now he must return quickly to the defence |  |
|  |  |  |  |  |  |  |  |  |  |
|  |  |  |  |  |  |  |  |  |  |
|  |  |  |  |  |  |  |  |  |  |
| **M2** | **Sequences** | **Context Objective** | **RW** | **RB** | **HC** | **P** | **LB** | **LW** |  |
| **ATTACK 6** | A6.1 | HC recovers the ball after an off-keeper. The wingers are quickly back to their corner to corner. P short to the line of 6m to position themselves. HC part in the race quickly, makes a pass at RB moving forward in the area of attack. | I'm running faster towards my wing **(A)** to dismiss the defence **(G)** | Here, I leave very quickly **(A)** to put myself back in front **(G)** and I request a pass from HC **(A)**, it sends me to the ball **(I)**  I see HC advance **(I)** | The goalkeeper recovers the ball **(I)**, it makes me a pass **(I)**, I see that RB part in the race quickly moving forward in the line of 9m **(I)**, I give him a high-pass **(A)** so that it goes to the pump **(G)** | I hurried in the defence zone **(A)** prior to the retreat of the defenders **(I)** and I keep my position **(I)** to provide a solution that goes to my partner, **(G)**  I put my arms up in the air **(A)** to attract the attention of RB **(G)**, RB doesn't see me **(I)**. | I'm in the race **(A)** to play the rise of fast ball **(G)** | I'm going to quickly put myself in front of **(A)**, I am preparing to receive a future ball **(EX)** |  |
|  | A6.2 | RB returns to HC which is advancing rapidly dribbling down the line of 6m. P is marked by two defenders. HC passes the ball P. P is found blocked by his opponent. The coach blows the whistle. | I position myself in the corner of the field **(A)**  P, it is located in a deadlock | I see the decline of defence quickly made **(I)**, I need to stalling, **(G)**, so I'm making a pass at HC **(A)** that happens to the area of 9m **(I).** | RB gives me the ball **(I)**,  I find myself annoyed by the two defenders **(I),** I would like to make a pass to P **(EX)**, I throw the ball to P **(A)**  Oh dear...a fault **(I)** | As usual I positioned on the area of 6-9m **(A)**.  I see HC forward with the ball **(I)**, I have the arm in front **(I)**, I expect a pass HC **(EX)**, she is frustrated by the opponent **(I)**, it is a mistake **(I)**  Here, I would have loved to be available for HC | When HC is moving forward with the ball **(I)**, I advance in parallel with **(A)**, then I prepare myself to have a pass HC **(EX)**.  Fault **(I)** | I look at the movement of the ball of my wing **(A)**  I see that LB advance **(I)** and preparing **(I)** to receive a ball of HC **(G)**.  But, the adversary has intercepted the ball **(I)**  P has not responded well to this claim, it remains idly |  |
|  |  |  |  |  |  |  |  |  |  |
|  |  |  |  |  |  |  |  |  |  |
| **M2** | **Sequences** | **Context Objective** | **RW** | **RB** | **HC** | **P** | **LB** | **LW** |  |
| **ATTACK 9** | A9.2 | HC made a feint to pass his direct opponent and dribbling moving forward in the area of defence. P makes a gesture with his hand. It is between two defenders from the opposing. HC makes a pass high in P. The ball is lost. | Here, I is not very involved **(I)**, I just need to watch the game **(A)** | J’expect that HC would make me a pass **(EX)** but he don’t see me **(I)**, I see that it plays only **(I)**  The pass-to-P is incorrect **(I)** , it was necessary for HC to make a pass to LB for that it can advance the ball **(G).** | LB makes me a pass **(I)**, I’m moving forwards with dribbling **(A)** to the line of 6m **(I)**, I find myself surrounded by three defenders **(I)**, I am looking for a partner **(A)** and there I throw the ball to P **(A)** | I see HC advance **(I)** with the ball **(I)**, I am preparing to have a pass **(EX)**, and as I find my self blocked **(I)**, I can't can’t get the ball back **(I)**, he throws me **(I)**, one of the opponents pushes me **(I)**, we lose the ball **(I)** | I make a simple pass to HC **(A)**  I wait until I redid the password **(EX)**  HC reacts quickly here **(I).** | Here, I'm not doing anything **(I)** |  |
|  |  |  |  |  |  |  |  |  |  |
| **MATCH 3** | | | | | | | | | |
|  |  |  |  |  |  |  |  |  |  |
| **M3** | **Sequences** | **Context Objective** | **RW** | **RB** | **HC** | **P** | **LB** | **LW** |  |
| **ATTACK 1** | A.1.4 | RB stalling the game by dribbling, he passes the ball to HC, which is close to the line of 6m and makes a pass to P. P find the space and pulls. Goal. | I'm waiting for a pass from HC **(EX)** , and then RB **(EX)** , but no one see me **(I)**, so I stay on my wing quiet **(I)** | I retreat to the area of 9m **(A)** and after I advance with HC **(A)** | LB makes me the pass again **(I)**, I do a little turn on the left **(A)**, I advance toward the line of 6m **(A)** to go to the shooting **(G)**, I see a hole between two defenders **(I)**, hop I make a pass to P **(A)** | My goal is to release me from my defenders **(G).** When HC advance to the line of 6m **(I),** I am ready for him to offer a solution to pass **(EX)**.  HC sends me to the ball **(I)**, I find myself enface of the cage **(I)**, I will tire **(A)**.  Enfin I score a goal **(I)** | I am doing a dribble **(A)** and I decline ine **(A)** to the area of 9m **(I)**, I pass the ball to HC **(A)** | It is true that I am not very involved in this attack, but my partners, they play well |  |
|  |  |  |  |  |  |  |  |  |  |
|  |  |  |  |  |  |  |  |  |  |
| **M3** | **Sequences** | **Context Objective** | **RW** | **RB** | **HC** | **P** | **LB** | **LW** |  |
| **ATTACK 5** | A.5.3 | HC leave quickly and advance into the area of defence, is blocked by a defender. HC does a rebound to the RB, which deviates to the left. A slight acceleration of RB. RB sends the ball to LB. LB is blocked by two defenders, he tries to give back the ball to RB. LB is pushed by one of the defenders. He falls. The coach blows the whistle. | I'm running the game **(A)** | Here, I see HC advance **(I)**, so I am preparing to be a future ballon **(EX),** suddenly HC makes a pass back **(I)**.  Coach always advises us do not do this pass, I recovers the ball hard with my left hand **(A),** in this moment, I don't know exactly what I go to do  I make a pass to LB **(A)** | I receive the ball from RB **(I)**, I’m running **(A)** to the defence zone **(I)**, I'm trying to find solutions **(G)** and gooing to the tire **(G)**, I find the two opponents in front of me **(I)**, the defence hinders me to move forward **(I)**.  RW does not move **(I)**, I have not found a solution **(I)** to make a pass back **(A)**, I throw the ball **(A)** | I try always try to be available for my partner **(EX)** in order to offer them solutions **(G)**  I try to take my defenders with me **(EX)** in order to create space for my partner,**(G)** | When RB receives the ball **(I)**, I think he's going to advance into the line of 6m **(EX)**, therefore, there will be no more opportunity to pass **(I)**  Of the sudden, I get a pass unexpected **(I)**, I advance **(A)**, I find before me a block of defenders **(I)** I am stopped by two opponents **(I)**, I try to make a pass **(G),** I fall **(A)** | I look at the movement of the ball **(A)**  ALD is not there at all **(I)** |  |
|  |  |  |  |  |  |  |  |  |  |
|  |  |  |  |  |  |  |  |  |  |
| **M3** | **Sequences** | **Context Objective** | **RW** | **RB** | **HC** | **P** | **LB** | **LW** |  |
| **ATTACK 6** | A.6.3 | RB make a dribble, moving forward in the area of 6-9m. It is blocked by a defender. RB makes a pass to HC. The ball is intercepted by a defender. The ball is lost. | There, it misses the ball **(I)** | I am doing a dribble **(A)** and I put **(A)**, I find before me an opponent **(I)** that blocks me **(I)**, I try to make a pass to HC **(G)**, unfortunately, an adversary intercepte the ball **(I)** | RB make a dribble **(I)**, it makes me a pass **(I)**, an opponent intercepte the ball **(I)**, we lose the ball **(I)**  RB always done a single dribble, where he is forced to make a pass immediately, Ouuf his work has always bugged me a lot, and as I am the leader of the game, I advise him ‘many times’ to avoid this method, because it means we lose the ball, but unfortunately, as I didn't say anything. | RB advance towards the area of 6-9m **(I)**, I am preparing to offer a solution password **(G)** I see that he passes the ball back and forth **(I)**  I’would have liked me to do this, because I had the opportunity to go to the pump  Oh, we loses the ball! **(I)** | RB has not played well, he always makes us lose the ball with this work | RB repeat the same mistake **(I)** |  |
|  |  |  |  |  |  |  |  |  |  |
|  |  |  |  |  |  |  |  |  |  |
| **M3** | **Sequences** | **Context Objective** | **RW** | **RB** | **HC** | **P** | **LB** | **LW** |  |
| **ATTACK 7** | A.7.3 | LW is blocked by two defenders, redid the password to LB. LB runs fast and advance to the line of 6m and tries to make the tire. LB is stopped by a defender and falls. The coach blows the whistle. Fault. | I am far from the action **(I)**.  I look at the progress of the game **(A)** | I follow the movement of the ball **(A)** | I look at the game **(A)**  I did not understand LB, that is what he will do. | I am running the game **(A)**  LB advance to the line of 6m **(I)**, I don't know exactly what to do.  I think that LB completes the action alone **(EX)** | LW returns me the ball **(I)**, then I've got to move on down the line of 6m **(G)**.  I found two defenders keep me moving forward **(I)**, I expect that P is involved **(EX)**, to support me **(G)** one of the two opponents are pushing me **(I)**, I fall **(A)**.  We lose the ball | Receiving the ball **(I)**, I find it blocked by two defenders **(I)**, I try to find a solution password **(A)**, and I pass to LB **(A)** |  |
|  |  |  |  |  |  |  |  |  |  |
|  |  |  |  |  |  |  |  |  |  |
| **M3** | **Sequences** | **Context Objective** | **RW** | **RB** | **HC** | **P** | **LB** | **LW** |  |
| **ATTACK 9** | A.9.3 | HC makes a pass to LB that part in the race moving forward in the line of 6m. LB is stopped by the two opponents. The ball is lost. | I can't do anything.  I look at the game **(A)** | I'm trying to watch the game **(A)** | I send the ball to LB **(A).**  I advance with LB **(A)** to provide a solution of the forward pass **(G)** | I am marked by two opponents **(I)**, and I watch carefully the progress of the game **(A)** | I receive the ball from HC **(I)**.  I advance quickly towards the center **(A)** I doing a dribble **(A)**, to go to the tire **(G)**, I find myself blocked by defenders that are grouped **(I)**,the ball is lost **(I)** | I find myself far from the action **(I)** |  |
