# Supplementary material for: Influence of diurnal variations on cognitive coordination and misunderstanding in elite male handball players
Source: PeerJ. 2026 Jan 15;14:e20370. doi: 10.7717/peerj.20370 (PMC12812274; doi:10.7717/peerj.20370)
Supplement: Supplemental Information 3 — Detailed verbalization analyses from player interactions during handball match sequences across three time points (10:00, 14:00, and 18:00). Includes the coding of player utterances based on four components of the Recognition-Primed Decision (RPD) model: Actions (A), Relevant Clues (I), Plausible Goals (G), and Expectations (EX). It identifies instances of misunderstanding and contradictory forms of shared cognition, the degree of coordination, and the sharing modes. Figures 2 and 3 provide quantitative summaries of the frequency and temporal variation of these elements. [file peerj-14-20370-s003.docx]

**MATCH 1**

| **Attack** | **Sequences** | **Player**  **involved** | **Preview of Verbalization** | **Number**  **of players involved** | **Actions**  **(A)** | **Relevant clues**  **(I)** | **Plausible**  **Goals**  **(G)** | **Expectations**  **(EX)** | **Contained typical shared** | **Forms typical deducted** |
| --- | --- | --- | --- | --- | --- | --- | --- | --- | --- | --- |
| **Attack 1** | **A1.2** | **HC :**  **LW :** | I'm doing a dribble **(A)** and I advance toward the area of 6-9m **(A)**, I want to give the ball to P **(EX),** I find myself stuck **(I)**we played two back **(I)**, I try to make a pass at LW **(EX),** I see LW is far from the action **(I)**, I turn on the right **(A)** to look for another partner **(G)**, I advance **(A)**, then I make a pass to RB **(A)**.  I see RB is not free **(I)**, it is surrounded by four defenders **(I)**, I step back towards the median line **(A)** I try to provide a solution to pass **(EX).**  I look for what is happening **(A),** I see HC is blocked **(I)**, I go out of my wing **(A)**, I try to help him **(EX)**, then I move **(A)** for him to offer a solution of **(G)**,... , **HC** no longer see myself **(I),** he gives the ball to RB **(I).** | 2 | Not  coordinated | Not  shared | Divergent | Not  listed | C6 | Misunderstanding |
|  |  |  |  |  |  |  |  |  |  |  |
| **Attack 2** | **A2.3** | **HC :**  **RB :**  **RW:** | I have the ball **(I)**, I digress to the right **(A)**, I want to give the ball to RW (**EX)** but I find myself blocked by two defenders **(I)**, as a result, RB is near me **(I),** I advance **(A)** and I can't find another solution **(I)** that of him make a pass **(A).**  HC deviates on the right **(I)**, it aims RW in its visual field **(I)** and suddenly I see that I excluded from this action **(I).**  I get a pass unexpectedly HC **(I)**, I go straight to RW **(A).** I step back three steps **(A)** pending the happening of RW **(EX)** but unfortunately RW is beyond me **(I).**  RB sends me to the ball **(I)**, I is marked by an opponent **(I)**, I move **(A),** I am headed to the line of 9m **(A)**, to share the ball with HC **(G)** , and then I give him a high-pass **(A).** | 3 | Not  coordinated | Not shared | Divergent | Divergent | C5 | Misunderstanding |
|  |  |  |  |  |  |  |  |  |  |  |
| **Attack 3** | **A3.3** | **HC :**  **RB :**  **P :** | I'm leaving in the race to the zone 6-9m **(A)** to go to the shooting **(G)**, I find myself blocked by a defender **(I)**, I make a pass at RB **(A)**.  I'm waiting for a pass from RB **(EX),** I put my hands in front **(A)** to provide a solution that password **(G)**, as a result, RB, reacting without thinking **(I)**.  We lose the ball **(I).**  We start the work with my partners in cooperation and then each one of us wants to show  HC sends me to the ball **(I)**, I make a feint to my direct opponent **(A)**, I digress on the left **(A)**, I try to pass the ball to HC **(G)**, I find myself blocked by two defenders **(I)**, so I'm making a pass to P **(A).**  I think we found a quick solution **(EX)**, But unfortunately, it was not successful **(I)**, we lose the ball **(I)**.  As usual, I position myself on the defence area **(A)**, marked by two defenders **(I)** , waiting for an opportunity to exchange with my partners **(EX)**.  I am the movement of the ball by paying attention to the rear **(A)** that could send me to the ball **(EX)**.  As I find myself surrounded by three defenders **(I),** I never expected a pass from RB **(I)**. I'm not available **(I)**.  All of a sudden, I made a low pass **(I).** Simply, RB does not know what to do. | 3 | Not  coordinated | Shared | Divergent | Divergent | C7 | Contradictory |
|  |  | **RB :**  **LB :** | HC sends me to the ball **(I)**, I make a feint to my direct opponent **(A)**, I digress on the G **(A)**, I try to pass the ball to HC **(G)**, I find myself blocked by two defenders **(I)**, so I'm making a pass to P **(A)**  I think that there is a quick solution **(EX)**, But unfortunately, it was not successful **(I)**, we lose the ball **(I)**.  I am prepared to receive the ball **(EX)**.  I'm waiting for RB to pass **(EX),** and I will raise my hands **(A)** to attract his attention, **(G)** and offer him a passing option **(G),** but to no avail**.** I'm getting a bit fed up | 2 | Not  coordinated | Not shared | Divergent | Divergent |  | Misunderstanding |
|  |  |  |  |  |  |  |  |  |  |  |
| **Attack 4** | **A4.1** | **RW :** | I recovers the ball **(A)**, I try to go more quickly **(A)** to realize the rise of fast ball **(G)**, the decline of the opposing defense is done quickly **(I)**, of a sudden, RB is a bit off **(I)**, I stalling speed **(A)** in order to seek a partner **(G)**, I step back to make a pass at RB **(G)**.  I gives the ball to RB **(A)**, I start running towards my wing **(A)** , and I expect that I redid a pass **(EX)** | 2 | Not  coordinated | Shared | Divergent | Divergent | C1 | Contradictory |
|  |  | **RB :** | RW part in the race **(I),** I would like to leave quickly **(EX)** for it to support **(G)**, but I think he has advanced compared to me **(I)**, of a sudden, I expect it to go to the tire **(EX),** I'm trying to I accelerate to receive a ball **(G)**.  RW makes me a pass **(I)**, I make a dribble **(A)**, I digress to the G **(A)**, I find myself blocked by two defenders **(I)**, I see ARG free **(I)**, I give him a pass **(A).** |  |  |  |  |  |  |  |
|  |  | **RB :** | RW part in the race **(I),** I would like to leave quickly **(EX)** for it to support **(G).**  RW is advanced compared to me **(I)**, as a result, I expect it to go to the tire **(EX)**, even when I try I accelerate to receive a ball **(G)**  RW makes me a pass **(I)**, I make a dribble **(A)**, I digress to the left **(A)**, I find myself blocked by two defenders **(I)**, I see LB open **(I)**I give him a pass **(A).** | 2 | Not  coordinated | Not - shared | Not  listed | Divergent |  | Misunderstanding |
|  |  | **HC :** | I would like to run quickly **(EX)** to receive a ball **(G).**  I am waiting of RB to make me a pass **(EX)**, I digress on the right **(A)**, I put my hands in front **(A)**, RB crosses his race in front of me , without any regard **(I).** |  |  |  |  |  |  |  |
|  | **A4.2** | **LW :**  **LB :** | I put on my wing **(A)**, I expect that LB sends me the ball **(EX)** since I had enough space on the line 6m **(I).**  LB can't see myself at all **(I).**  I get the ball from RB **(I)**, I go in the race moving forward in the line of 6m **(A)**. Two defenders are in front of me **(I)**, RB calls me **(I)**, I must be going in the tire only **(G)**, I jump **(A)** and I take **(A)**, not goal **(I).** | 2 | Not  coordinated | Not shared | Divergent | Divergent | C6 | Misunderstanding |
|  |  | **RB :**  **LB :** | I expect that LB I redid the password **(EX)**, I put the hands in the air **(A).**  LB plays alone **(I)** and pulls **(I).** The action of pulling of LB is unexpected**.**  I get the ball from RB **(I),** I go in the race moving forward in the line of 6m **(A).**  Two defenders are in front of me **(I),** RB calls me **(I),** I have to go there all alone **(G),** I jump **(A)** and I take **(A),** not goal **(I).** | 2 | Not  coordinated | Shared | Divergent | Divergent |  | Contradictory |
|  |  |  |  |  |  |  |  |  |  |  |
| **Attack 5** | **A5.1** | **HC :**  **RB :**  **P :**  **LB :** | I get the ball RB **(I)**, I advance toward the line of 9m **(A)**  I found a defender in front of me **(I)**, I try to exceed, to go to the tire **(G)**, it prevents me to move forward **(I).**  The coach blows the whistle **(I)**  I see HC is blocked by a defender **(I)**, I expect that I am to make a pass **(EX),** I lift up my left hand **(A)** to have a ball **(G)**, but HC back is turned **(I)** and headed to the left **(I)**.  I am in the defence zone between the two opponents **(I)**.  I'm waiting for a pass from HC **(EX)**  I try to be available to HC **(G)** in order to provide a solution that password **(G)**, but he doesn't see me **(I)**.  The coach blows the whistle **(I)**.  I am prepared to receive the ball **(EX),** I put my arms forward **(I)**. I see that HC is looking to surpass his opponent **(I).** Then, I step back **(A)**. | 4 | Not  coordinated | Not shared | Divergent | Divergent | C5 | Misunderstanding |
|  | **A5.2** | **LB :**  **P :**  **HC :** | I meet with HC **(A).** He gives me the ball **(I)**, I try to give it back **(G)**.  Since it is a little decreased **(I)**, I am preparing to advance into the line of 6m **(G).**  I am blocked by three defenders **(I)**, I make a feint to exceed **(A)** , and then I'm doing a low pass to P **(A)**  I get the ball **(A)** and I go to HC **(A)** to re-start the game **(G)**. Then, I go back on the line 6m **(A)**  I stay on my position **(EX)**  Despite that, I find myself blocked by three defenders **(I)**, LB I made a low pass **(I)**, I try to recover **(G)** but I have not been able to **(I)**. An opponent pushes me **(I)**, the ball is lost **(I).**  I am preparing to announce a combination **(G)** but ARG upsets all the work **(I)**.  I crossed my race with LB **(A)**, I place myself on the left **(A).**  I'm waiting for a pass from LB **(EX)**, I must prepare myself to help him **(G).**  LB continues to play all alone **(I)**.  The ball is lost **(I)** | 3 | Not  coordinated | Shared | Divergent | Divergent | C6 | Contradictory |
|  |  |  |  |  |  |  |  |  |  |  |
| **Attaque 6** | **A6.2** | **RB :** | I have the ball **(I)**, I try to make a pass to HC **(G)**, I see a defender who is close to him **(I)** and as a result, P comes out of the box 6-9m to support me **(I)**, and I give him a pass **(A).**  P advance to the line of 6m **(I)**, when he takes the position of jump shooting **(I)**, I expect it to go to the tire **(EX)**. P me makes a pass unexpected **(I)**.  The ball drops **(I)**, I get it **(A)** and I will pass directly to RW **(A)**  I see RB surrounded by two defenders **(I)**, so I'm leaving the defence area **(A)** to provide a solution that password **(G).**  I get the ball **(I**), I find before me three defenders **(I)**, so I gives him the ball **(A)**  I lift up my hand **(A)** in order to attract the attention of RB **(G)** and that it will make me a pass **(G)**, the defenders are distant **(I),** I would like to go to the tire **(EX)**, no one see me **(I).** | 3 | Not  coordinated | Not shared | Divergent | Divergent | C5 | Misunderstanding |
|  |  | **P :**  **LB** |  |  |  |  |  |  |  |  |
|  |  |  |  |  |  |  |  |  |  |  |
|  | **A6.4** | **RB :** | I get the ball from P **(I)**, I try to give back to HC **(G).** I'm ready to go pull **(EX)**, I make a feint **(A)** to switch my direct opponent **(G)** and I shoot from the line of 9m **(I)**. | 2 | Not  coordinated | Shared | Divergent | Divergent | C4 | Contradictory |
|  |  | **HC :** | Here, I am announcing Yougo **(A)**, LB passes me the ball **(I)**, I dribble **(A).** I look **(EX)** as P arrives and crosses behind me **(I),** I gives him the ball **(A)**. I meet with LB **(A).**  I'm waiting for a pass from ARB **(EX)**, so I prepare myself to go in the race **(G)** and exchange the ball with him **(G)**. I see that RB plays alone **(I)** |  |  |  |  |  |  |  |
|  |  | **RB :**  **LB :** | I get the ball P **(I)**, I try to give back to HC **(B).** I'm ready to go pull **(EX)**, I dribble **(A)**, I accelerate **(A)**, I make a feint to pass my direct opponent **(A)** and I take **(A)** the line-of-9m **(I)**.  Ai, ai, ai... I miss my chance **(I)**  I see the announcement of Yougo **(I),** I lift my hands **(A)** I request the ball **(A)**, no one sees me **(I)**, I'm fed up in attack.  I still have the same problem with the RB, it is in disagreement. | 2 | Not  coordinated | Not shared | Not  listed | Divergent |  | Misunderstanding |
|  |  |  |  |  |  |  |  |  |  |  |
| **Attack 7** | **A7.2** | **LB**  **HC:** | I advance towards the area of 6-9m **(A)**, I find before me a defender who is preventing me from getting to the line 6m **(I)**, so I do not need a pass to HC **(G).**  In this action, there was a misunderstanding with HC, it was intended to play Yougo **(I)**, we are going to repeat again the action.  LB sends me to the ball **(I)**, I try to recover **(EX)** so I have a screen delay **(I)** and I missed the ball **(I).** | 2 | Not  coordinated | Shared | Divergent | Not  listed | C5 | Contradictory |
|  |  | **RB :** | I get the ball to the LB **(I)**. I am preparing to go to the tire **(G)**, so I advance **(A).** I find myself blocked by three opponents **(I)**, so I throw the ball on the left **(A)**. Fortunately, LB get **(I).** | 2 | Not  coordinated | Not shared | Divergent | Divergent |  | Misunderstanding |
|  |  | **HC :** | LB sends me to the ball **(I),** I try to recover **(EX),** so I have a screen delay **(I)** and I missed the ball **(I).** Here, I'm waiting for a pass from RB **(EX),** so I'm preparing for the receive **(EX),** of a sudden, he throws the ball without any reflection**. (I).** It amazes me. I fail to understand my partners back (RB, LB)**.** |  |  |  |  |  |  |  |
|  | **A7.3** | **LB :**  **HC :** | I recovers the ball **(A)**, I dribble **(A)**, LW is near me **(I)**, I give him a pass (**A).**  I 'm preparing for a pass from LB for the second time **(EX)**, I have the arm in front **(I)**, LB made a feint of pass **(I)**, I feel that it is blocked **(I).** LB goes to LW **(I)**.  Finally**,** LW makes me a pass **(I)**, we run the ball **(A)** before that I announce **(A)** what we must play in attack **(I)**. | 2 | Not  coordinated | Shared | Not  listed | Divergent | C3 | Contradictory |
|  |  | **RB :**  **LB:** | I will lift my hands high **(A)** to attract the attention of LB **(B)**, but LB don't see me **(I)**. He decides to play alone **(I)**.  I'm running the ball with HC and LB in the area of 9m **(A).**  I get the ball **(A)**, I dribble **(A)**, LW is near me **(I)**, I give him a pass (**A).**  LW does not understand what is what are we going to play now, I would like to if it remains on its wing quietly **(EX),** it's going to cause us discomfort at the level of displacement. | 2 | Not  coordinated | Not  shared | Divergent | Not  listed |  | Misunderstanding |
|  | **A7.5** | **P :**  **RB:** | I see RB has the ball **(I)**, I lift up my hand **(A)** for me to make a pass **(G)** so that I could go to the tire **(G)**.  RB make a passe for LB **(I)**. For the second time **(I)**, I lift my two hands, **(A)** in order to attract the attention of RB **(B)**, RB continues to play all alone **(I)**. RB misses to score **(I)**  I get the ball of HC **(I)**, I digress on the left **(A)**, I'm doing a dribble by pushing to LB **(A)**, I give him a pass **(A).** | 2 | Not  coordinated | Not  shared | Divergent | Not  listed | C6 | Misunderstanding |
|  |  |  |  |  |  |  |  |  |  |  |
|  |  |  |  |  |  |  |  |  |  |  |
| **Attack 8** | **A8.1** | **RB :**  **LB:** | If LB advance to the line of 6m **(I)**, I lift up my hand **(A)** so that it sends me the ball **(G)**, because I find myself in a good position **(I)** to go to the tire **(G)**, LB don't see me **(I)**  I get the ball from LW **(I),** I advance towards the area of 6-9m **(A),** I dribble **(A),** I am looking for a position **(A)** to penetrate between the defenders on the line of 6m **(G),** I find myself blocked by two opponents **(I).** I try to make a pass at DC **(EX),** but I can't **(A).** | 2 | Not  coordinated | Not  shared | Divergent | Not  listed | C6 | Misunderstanding |
|  |  | **HC :**  **LB :** | I see that LB has the ball **(I)** and advance to the defence zone **(I)**, I go in the race at the same time **(A)**, I expect it will give me a pass **(EX)** to move forward **(G)** and go to the pump **(G)**, it is a failure by the defendant **(I)**. Fault (**I)**.  Here, Iexpect that HC deviates on the left **(EX)** but it stays in the center **(I)**, the ball is lost **(I).** | 2 | Not  coordinated | Shared | Not  listed | Divergent |  | Contradictory |
|  |  |  |  |  |  |  |  |  |  |  |
| **Attack 9** | **A9.1** | **LB :**  **HC :** | I get the ball **(A)**, I try to find a solution **(G),** I foresee that I could go to pull **(EX)**, I began to dribble forward **(A)** to the line of 6m **(I)**, there are three opponents that I block **(I)**, I try to move forward **(EX)** and then I do a high pass HC **(A).**  Here, HC remains on its position **(I)** instead of spreading on the left. I am waiting for the crossover **(EX)**,  I see LB plays very quickly without sharing **(I)** as usual. I'm waiting for a pass of his hand **(EX).** But, It plays only **(I)**, it really amazes me.  LB is blocked **(I)**, finally, it makes me a pass **(I)**, I find before me four opponents **(I)**, I make a feint **(A)** to pass the opponents **(G)**, I turn to the right **(A)**, I pass the ball to RB **(A)** | 3 | Not  coordinated | Not shared | Divergent | Divergent | C7 | Misunderstanding |
|  |  |  |  |  |  |  |  |  |  |  |
|  |  | **P:** | I keep a good position **(I)** in order to be available for LB **(G).** Je plan to have a password LB **(EX)** and give it as a solution of passes **(G).**  LB continues to play alone **(I).** |  |  |  |  |  |  |  |
|  | **A9.2** | **LW:**  **LB:** | I put myself on the left wing **(A).**  The defenders are distant **(I)**, there is an opportunity of draws **(I)**, I call LB a lot of time **(A)** to have a pass **(G)**, and provide a solution **(G)** , but unfortunately, his position did not allow him to make me a pass **(I).**  I get the ball of HC **(I)**, I may give directly **(A)**, HC I redid the ball **(I).**  Here, I would like to make a pass to LW **(EX)**, but as I find myself placed in the wrong position **(I)**, so I'm doing a high pass at RB **(A)**. | 2 | Not  coordinated | Shared | Not  listed | Divergent | C3 | Contradictory |
|  | **A9.3** | **RB :**  **LB:**  **HC :** | HC sends me to the ball **(I)**, I wish to make a pass to P **(EX)** which is located on the line of 6m **(I)** and as I am blocked by two defenders **(I)**, I send the ball to HC **(A).**  I expect that DC gives me back the ball **(EX).**  I am preparing to receive a ball of HC **(EX)**, I advance a step **(A)** until a pass **(EX),** and then I see HC advance to the line of 6m quickly **(I)**, I’am preparing to the tire **(I).**  RB sends me to the ball **(I)**, I hope to go to the tire only **(EX)**, I argue **(A)** to the area of 6-9m **(I)**, I try to go to the fire **(EX)** then, I make a feint **(A)** to switch my direct opponent **(G)** and I pull **(A)**.  Unfortunately, I missed the goal **(I).** | 3 | Not  coordinated | Not shared | Divergent | Divergent | C5 | Misunderstanding |
|  |  |  |  |  |  |  |  |  |  |  |
| **Attack 10** | **A10.2** | **HC :**  **LB :**  **LW:** | I go to LB **(A)**, I wish that I hear the bullet **(EX)**, I see that LB plays without any coordination with me **(I)**, the same problem always with him, I rest in the center without moving **(EX).**  HC gives me the ball **(I)**, in that time, I am preparing to cross the block defence **(G)**, I find myself blocked by two defenders **(I)**, there is no space **(I)** to go shoot **(G)**, I would like to make a pass to P **(EX).**  When I see LB advance with the ball **(I)**, I prepare myself to have a pass **(EX).** LB don't see me **(I).**  I find myself alone **(I)**, there is space for away from here **(I).** | 3 | Not  coordinated | Not shared | Divergent | Divergent | C7 | Misunderstanding |
|  |  | **P :** | LB sends me to the ball **(I)** despite the fact that I'm not available **(I)**, I try to retrieve the ball **(G)**, the defender pushes me in the back **(I)**, the ball falls off **(I)**, it is a mistake **(I).**  There is no space **(I)** to go shoot **(G),** I would like to make a pass at P **(EX).** | 2 | Not  coordinated | shared | Divergent | Divergent |  | Contradictory |
|  |  | **LB :** |  |  |  |  |  |  |  |  |

| **Elements of the RPD Model (Figure 2)** | | | | |  |  |  |
| --- | --- | --- | --- | --- | --- | --- | --- |
|  |  |  |  |  |  |  |  |
| Forms | RPD elements | Match 1 (10h00) | | Match 2 (14h00) | | Match 3  (18h00) | |
|  |  | N | % | N | % | N | % |
| Contradictory | Actions (A) | 44 | (62,85%) | 19 | (27,14%) | 7 | (10,00%) |
|  | Relevant Clues ( C) | 71 | (68,93%) | 26 | (25,24%) | 6 | (5,82%) |
|  | Plausible Goals (G) | 26 | (68,42%) | 9 | (23,68%) | 3 | (7,89%) |
|  | Expectations (E ) | 19 | (82,60%) | 2 | (8,69%) | 2 | (8,69%) |
|  | | | | | | | |
| Misunderstanding | Actions (A) | 74 | (70,47%) | 17 | (16,19%) | 14 | (13,33%) |
|  | Relevant Clues ( C) | 111 | (65,29%) | 32 | (18,82%) | 27 | (15,88%) |
|  | Plausible Goals (G) | 34 | (72,34%) | 7 | (14,89%) | 6 | (12,76%) |
|  | Expectations (E ) | 39 | (78,00%) | 7 | (14,00%) | 4 | (8,00%) |


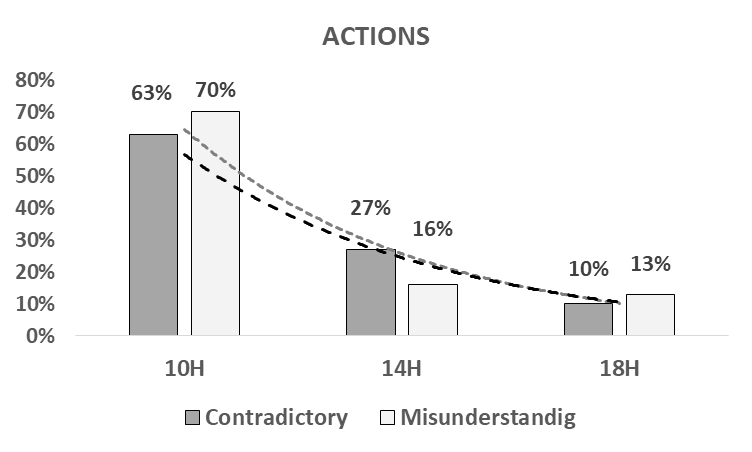

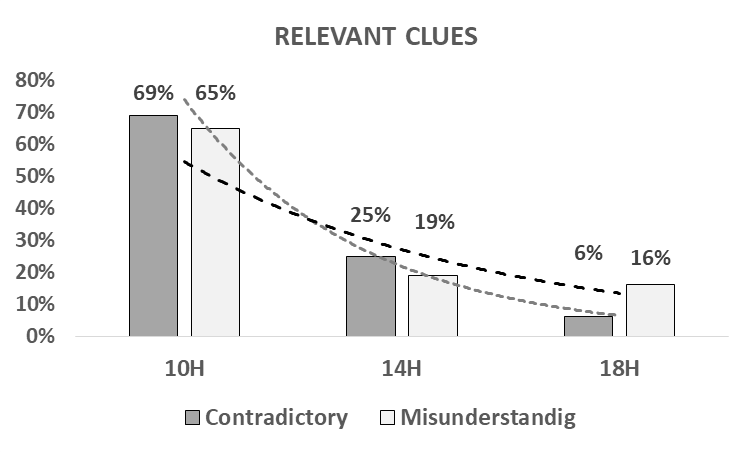


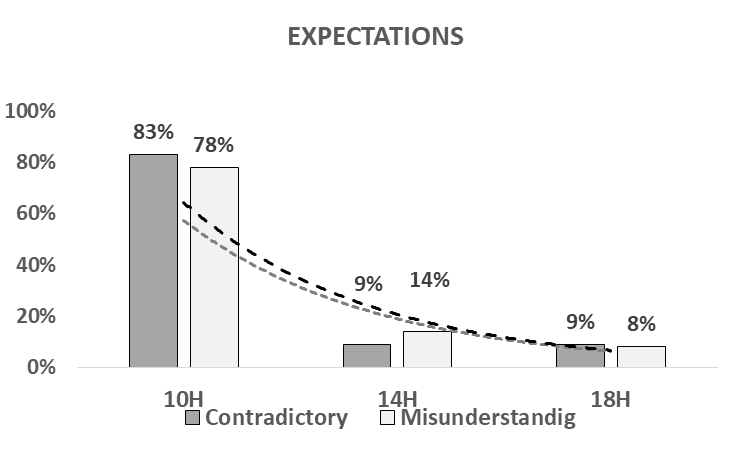

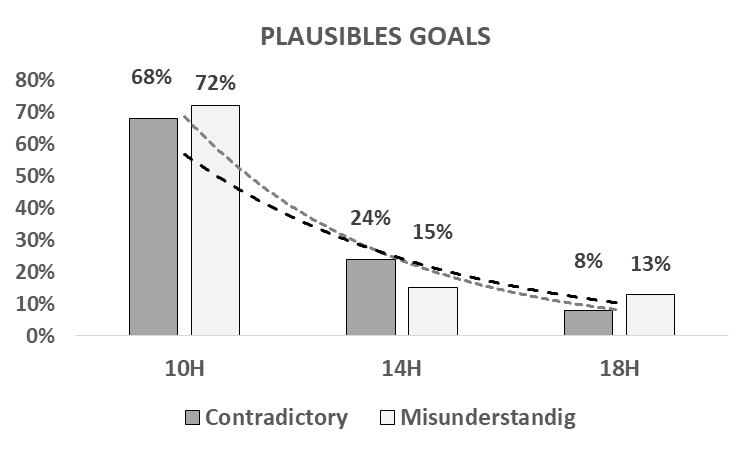


**Figure 2:** Diurnal variation of the elements of RPD model according to contradictory and misunderstanding forms.

| **Sharing modes of the contradictory and misunderstanding forms in Match 1 (10h00) (Figure 3)** | | | | | | | |
| --- | --- | --- | --- | --- | --- | --- | --- |
|  |  |  |  |  |  |  |  |
| Forms | | Match 1 (10h00) | | Match 2 (14h00) | | Match 3 (18h00) | |
|  |  | N | (%) | N | (%) | N | (%) |
| Contradictory | | 10 | (66,66%) | 3 | (20,00%) | 2 | (13,33%) |
| Misunderstanding | | 15 | (65,21%) | 5 | (21,73%) | 3 | (13,04%) |

**Figure 3:** Evolution of sharing mode of the contradictory and misunderstanding forms.

**MATCH 2**

| **Attack** | **Sequences** | **Player**  **involved** | **Preview of Verbalization** | **Number**  **of players involved** | **Actions**  **(A)** | **Relevant clues**  **(I)** | **Plausible**  **Goals**  **(G)** | **Expectations**  **(EX)** | **Contained typical shared** | **Forms typical deducted** |
| --- | --- | --- | --- | --- | --- | --- | --- | --- | --- | --- |
|  |  |  |  |  |  |  |  |  |  |  |
| **Attack 2** | **A2.1** | **HC:** | P recovers the ball **(I)** and makes me a pass **(I)**, I start running towards the front **(A)**, I go to RB **(A)** that the returns to RW **(I)**and then he gives me back the ball **(I)**, is made to circulate the ball **(A)** and I am preparing to announce a combination **(I).**  Here, I am announcing Yougo **(A)** with my hand almost three times **(I)**, but P doesn't see me **(I)**, his gaze is focused on ae something else **(I),** I try to find a solution **(EX)** | 2 | Not  coordinated | Not shared | Divergent | Divergent | C3 | Misunderstanding |
|  |  | **P :** | I go fast **(A)** to the defence zone **(I)** to place me near my defenders **(G)** and I keep my position **(I).**  I'm waiting for the announcement of HC **(EX).** |  |  |  |  |  |  |  |
|  | **A2.2** | **HC :**  **P :** | I am announcing again the combination Yougo **(A)** with my hand and my voice **(I)** to attract the attention of P **(G)** to facilitate the exchange of the ball **(G).**  I see P moves **(I)**  I am preparing **(A)** to throw the ball to P **(G)** , and then to cross the race with LB **(G)**  I see the announcement **(I)** and I hear the word ‘soon’ from HC **(I)**, and then he raises his hand **(I)**  I'm in the running **(A)** towards the zone of attack **(I)**, I crossed behind HC **(A)**, I expect the ball **(EX)**, I have a screen delay **(I)** and kick the ball drops **(I)**.  I can't seem to catch up with my hands **(A)**, I tried **(A)** I step back towards the median line **(A)** to recover **(G)**, a defender pushes me in the back **(I).**I fall **(A)** | 2 | Not  coordinated | Shared | Convergent | Not  listed | C4 | Contradictory |
|  | **A2.3** | **HC :**  **LB :** | I recovers the ball **(A)**, I begin to dribble **(A)** I advance **(A)** to the line of 6m **(I)**, and hop I make a pass to LB **(A)** that keeps a good position **(I)** to move quickly **(G)**, I don't know what he has, he can't catch the ball **(I).**  I see that HC has the ball **(I)**, I must prepare myself **(A)** to provide a solution that password **(G)**, I advance in the race **(A)** to attract his attention, **(G)**, it throws me the ball too behind **(I)**, I try to catch up **(A),** of a sudden, I have a screen delay **(I),** I miss the ball **(I).** | 2 | Not  coordinated | Shared | Not  listed | Not  listed | C5 | Contradictory |
|  |  |  |  |  |  |  |  |  |  |  |
| **Attack 3** | **A3.3** | **HC:**  **RB:** | I get the ball **(I)** and I’m advancing rapidly **(A),** I dribble **(A)**, hop, it was a defender in front of me **(I)**, my goal is to exceed **(B)**, so I'm making a feint **(A)** and I still cautious **(I).**  I advance toward the line of 6m **(I)** and I pull **(A)**.  Unfortunately, I completely missed my pulls **(I)**  When HC advance **(I)**, I crossed behind him **(A)**, I expect it will give me a pass **(EX**) to go to the tire **(G).**  I would have liked to finish the attack to the outside. | 2 | Not  coordinated | Not shared | Not  listed | Divergent | C5 | Misunderstanding |
|  |  |  |  |  |  |  |  |  |  |  |
| **Attack 6** | **A6.1** | **P :**  **RB:** | I go fast in the defence area **(A)** before the retreat of the defenders **(I)** and I keep my position **(I)** to provide a solution that goes to my partner, **(B)**  I put the arms up in the air **(A)** in order to attract the attention of RB **(G)**, RB doesn't see me **(I)**.  Here, I leave very quickly **(A)** for me to put in before **(G)** and I request a pass from HC **(A)**, it sends me to the ball **(I)**  I see HC advance **(I).** | 2 | Not  coordinated | Not shared | Divergent | Not  listed | C1 | Misunderstanding |
|  | **A6.2** | **HC :**  **LB :** | RB gives me the ball **(I),** I am embarrassed by two defenders **(I),** I would like to make a pass to P **(EX)**, I throw the ball to P **(A)**  Oh my...fault **(I)**  When HC is moving forward with the ball **(I)**, I advance in parallel with **(A)**, then I prepare myself to have a pass from HC **(EX)**.  Fault **(I).** | 2 | Not  coordinated | Not shared | Not  listed | Divergent | C6 | Misunderstanding |
|  |  |  |  |  |  |  |  |  |  |  |
| **Attack 9** | **A9.2** | **HC :**  **P :** | LB makes me a pass **(I)**, I argue **(A)** to the line of 6m **(I)** and I dribble **(A)**, I find myself surrounded by three defenders **(I)**, I am looking for a partner **(A)** and here I throw the ball to P **(A)** to advance **(G).**  I see HC advance **(I)** with the ball **(I)**, I prepare myself to have a pass **(EX)**, and as I am stuck **(I)**, I can't get the ball **(I)**, I, lance **(I)**, one of the opponents pushes me **(I)**, we lose the ball **(I).** | 2 | Not  coordinated | Shared | Not  listed | Divergent | C5 | Contradictory |
|  |  | **RB:** | I’expect that HC would make me a pass **(EX)** but he doesn't see me **(I)**, I see that it plays only **(I)**  The pass-to-P is incorrect **(I),** it was necessary for HC to make a pass at LB for it to move forward with the ball **(G)**  To the line of 6m **(I)** , I find myself surrounded by three defenders **(I)**, I throw the ball to P **(A)**  I am a simple password to HC **(A)**  I expect that I redid the password **(EX)**  HC reacts quickly here **(I).** | 3 | Not  coordinated | Not shared | Not  listed | Divergent |  | Misunderstanding |
|  |  | **HC:** |  |  |  |  |  |  |  |  |
|  |  | **LB :** |  |  |  |  |  |  |  |  |

| **Elements of the RPD Model (Figure 2)** | | | | |  |  |  |
| --- | --- | --- | --- | --- | --- | --- | --- |
|  |  |  |  |  |  |  |  |
| Forms | RPD elements | Match 1 (10h00) | | Match 2 (14h00) | | Match 3  (18h00) | |
|  |  | N | % | N | % | N | % |
| Contradictory | Actions (A) | 44 | (62,85%) | 19 | (27,14%) | 7 | (10,00%) |
|  | Relevant Clues ( C) | 71 | (68,93%) | 26 | (25,24%) | 6 | (5,82%) |
|  | Plausible Goals (G) | 26 | (68,42%) | 9 | (23,68%) | 3 | (7,89%) |
|  | Expectations (E ) | 19 | (82,60%) | 2 | (8,69%) | 2 | (8,69%) |
|  | | | | | | | |
| Misunderstanding | Actions (A) | 74 | (70,47%) | 17 | (16,19%) | 14 | (13,33%) |
|  | Relevant Clues ( C) | 111 | (65,29%) | 32 | (18,82%) | 27 | (15,88%) |
|  | Plausible Goals (G) | 34 | (72,34%) | 7 | (14,89%) | 6 | (12,76%) |
|  | Expectations (E ) | 39 | (78,00%) | 7 | (14,00%) | 4 | (8,00%) |


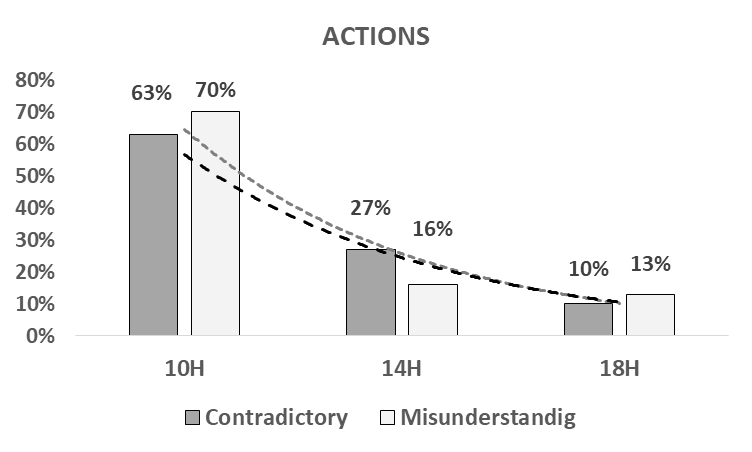

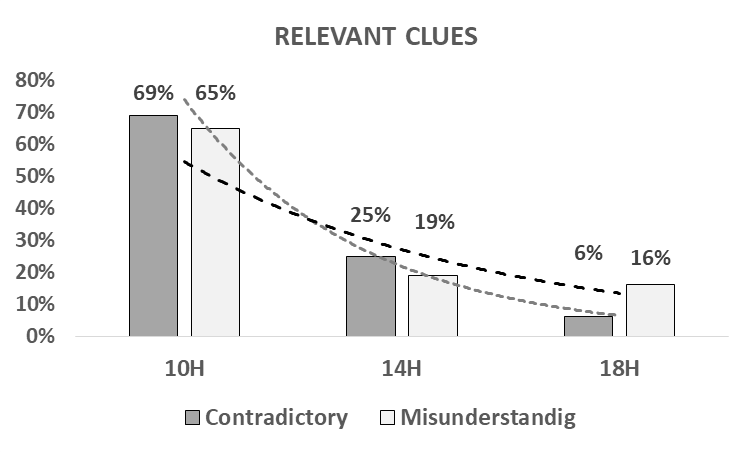


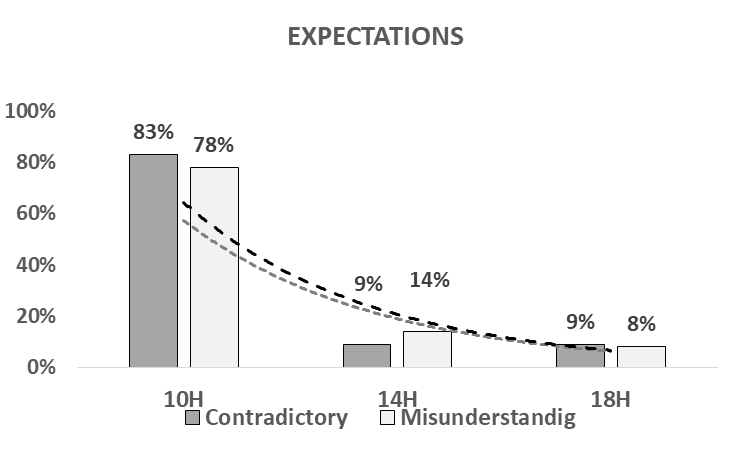

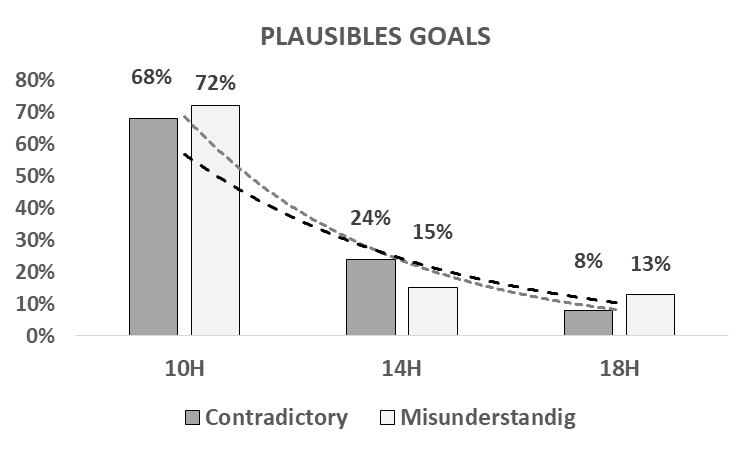


**Figure 2:** Diurnal variation of the elements of RPD model according to contradictory and misunderstanding forms.

| **Sharing modes of the contradictory and misunderstanding forms in Match 1 (10h00) (Figure 3)** | | | | | | | |
| --- | --- | --- | --- | --- | --- | --- | --- |
|  |  |  |  |  |  |  |  |
| Forms | | Match 1 (10h00) | | Match 2 (14h00) | | Match 3 (18h00) | |
|  |  | N | (%) | N | (%) | N | (%) |
| Contradictory | | 10 | (66,66%) | 3 | (20,00%) | 2 | (13,33%) |
| Misunderstanding | | 15 | (65,21%) | 5 | (21,73%) | 3 | (13,04%) |

**Figure 3:** Evolution of sharing mode of the contradictory and misunderstanding forms.

**MATCH 3**

| **Attack** | **Sequences** | **Player**  **involved** | **Preview of Verbalization** | **Number**  **of players involved** | **Actions**  **(A)** | **Relevant clues**  **(I)** | **Plausible**  **Goals**  **(G)** | **Expectations**  **(EX)** | **Contained typical shared** | **Forms typical deducted** |
| --- | --- | --- | --- | --- | --- | --- | --- | --- | --- | --- |
| **Attack 1** | **A1.4** | **RW**  **HC :**  **RB:** | I'm waiting for a pass from HC **(EX)** , and then RB **(EX)** , but no one see me **(I)**, so I stay on my wing quiet **(I).**  LB redid the password **(I)**, I make a small tower on the left **(A)**, I advance toward the line of 6m **(A)**, to go to the shooting **(G)** I see a hole between two defenders **(I)**, hop I make a pass to P **(A)**  I retreat to the area of 9m **(A)** and after I advance with HC **(A)** | 3 | Not  coordinated | Not  shared | Not  listed | Divergent | C6 | Misunderstanding |
|  |  |  |  |  |  |  |  |  |  |  |
| **Attack 5** |  | **HC :**  **RB :**  **LB :** | I get the ball from RB **(I)**, I **(A)** to the defence zone **(I)**, I try to find solutions **(G)** and go to the tire **(G)**, I find the two opponents in front of me **(I)**the defense annoys me to move forward **(I)**.  RW does not move **(I)**, I have not found a solution **(I)** to make a pass back **(A)**, I throw the ball **(A)**  Here, I see HC advance **(I)**, so I am preparing to be a future ballon **(EX),** the coup HC makes a pass back **(I)**.  The coach always advises us not to not to do this, I get the balllon difficult with the left hand **(A),** in that moment, I don't know exactly what to do  I make a pass to LB **(A)**  When RB receives the ball **(I)**, I think he's going to advance into the line of 6m **(EX)**, so there will be more of an opportunity to pass **(I)**  Suddenly, I get a password unexpected **(I)**, I argue **(A)**, I find before me a block of defenders **(I)**, I am stopped by two opponents **(I)**, I try to make a pass **(G),** I fall **(A)** | 3 | Not  coordinated | Not  shared | Divergent | Divergent | C7 | Misunderstanding |
|  | **A5.3** |  |  |  |  |  |  |  |  |  |
|  |  |  |  |  |  |  |  |  |  |  |
| **Attack 6** |  | **RB :**  **HC**  **PVT :** | I'm doing a dribble **(A)** and I put **(A)**, I find before me an opponent **(I)** that blocks me **(I)**, I try to make a pass to HC **(G)**, unfortunately, an opponent intercepte the ball **(I)**  RB doing a dribble **(I)**, it makes me a pass **(I)**, an adversary intercepts **(I)**, we lose the ball **(I)**  RB always done a single dribble, where he is forced to make a password immediately, phew his work has always bugged me a lot, and as I am the leader of the game, I advised him ‘not a lot of times’ to avoid this method because it makes us lose the ball, but unfortunately, as I didn't say anything.  RB advance towards the area of 6-9m **(I)**, I am preparing to offer a solution password **(G)**, I see that he passes the ball back and forth **(I)**  I liked that he could make me this, because I had the opportunity to go to the pump  Oh, one loses the ball ! **(I)** | 3 | Not  coordinated | Not  shared | Divergent | Not  listed | C6 | Misunderstanding |
|  | **A6.3** |  |  |  |  |  |  |  |  |  |
|  |  |  |  |  |  |  |  |  |  |  |
|  |  |  |  |  |  |  |  |  |  |  |
| **Attack 7** | **A7.3** | **LB :**  **P :** | LW I hear the ball **(I)**, then I advance to the line of 6m **(A)**.  I found two defenders keep me moving forward **(I)**, I expect that P is involved **(EX)**, to support me **(G)** one of the two opponents are pushing me **(I)**, I fall **(A)**.  Iam running the game **(A)**  LB advance to the line of 6m **(I)**, I don't know exactly what to do.  I think that LB completes the action alone **(EX)** | 2 | Not  coordinated | Shared | Divergent | Divergent | C7 | Contradictory |
|  |  |  |  |  |  |  |  |  |  |  |
| **Attack 9** | **A9.3** | **HC:**    **LB :** | I send the ball to LB **(A).**  I advance with LB **(A)** to provide a solution of the forward pass **(G)**  I get the ball of HC **(I)**.  I advance quickly towards the center **(A)**, I dribble **(A)** to go to the tire **(G)**, I find myself blocked by defenders that are grouped **(I)**, the ball is lost | 2 | Not  coordinated | Shared | Divergent | Divergent | C7 | Contradictory |
|  |  |  |  |  |  |  |  |  |  |  |
|  |  |  |  |  |  |  |  |  |  |  |

| **Elements of the RPD Model (Figure 2)** | | | | |  |  |  |
| --- | --- | --- | --- | --- | --- | --- | --- |
|  |  |  |  |  |  |  |  |
| Forms | RPD elements | Match 1 (10h00) | | Match 2 (14h00) | | Match 3  (18h00) | |
|  |  | N | % | N | % | N | % |
| Contradictory | Actions (A) | 44 | (62,85%) | 19 | (27,14%) | 7 | (10,00%) |
|  | Relevant Clues ( C) | 71 | (68,93%) | 26 | (25,24%) | 6 | (5,82%) |
|  | Plausible Goals (G) | 26 | (68,42%) | 9 | (23,68%) | 3 | (7,89%) |
|  | Expectations (E ) | 19 | (82,60%) | 2 | (8,69%) | 2 | (8,69%) |
|  | | | | | | | |
| Misunderstanding | Actions (A) | 74 | (70,47%) | 17 | (16,19%) | 14 | (13,33%) |
|  | Relevant Clues ( C) | 111 | (65,29%) | 32 | (18,82%) | 27 | (15,88%) |
|  | Plausible Goals (G) | 34 | (72,34%) | 7 | (14,89%) | 6 | (12,76%) |
|  | Expectations (E ) | 39 | (78,00%) | 7 | (14,00%) | 4 | (8,00%) |


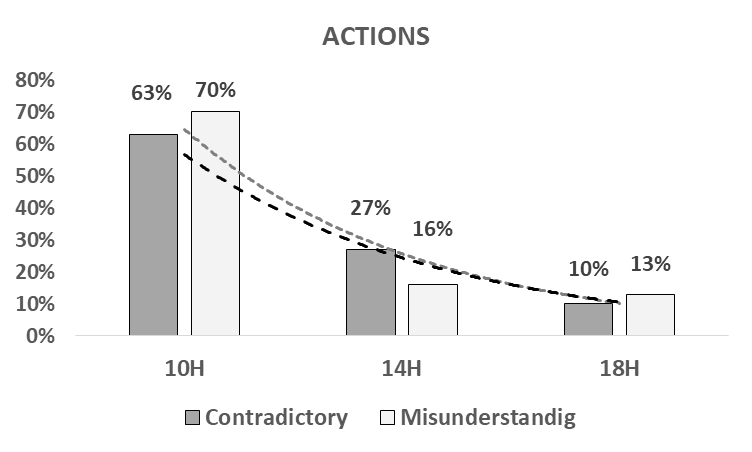

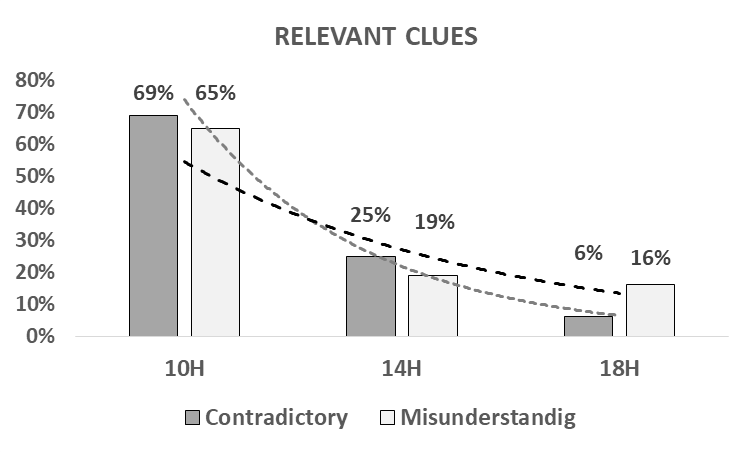


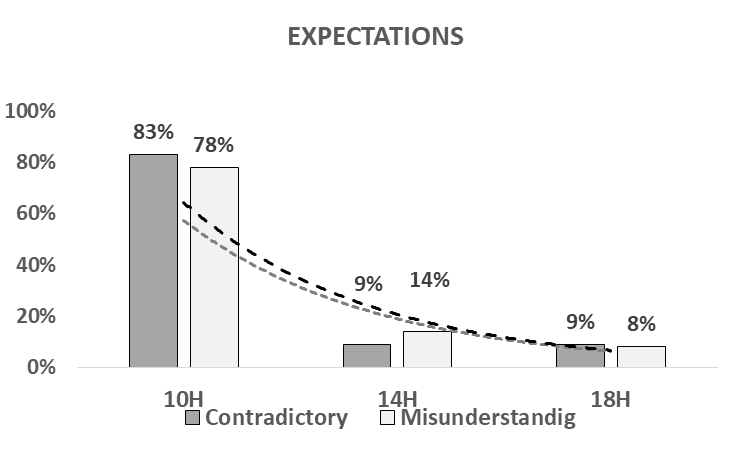

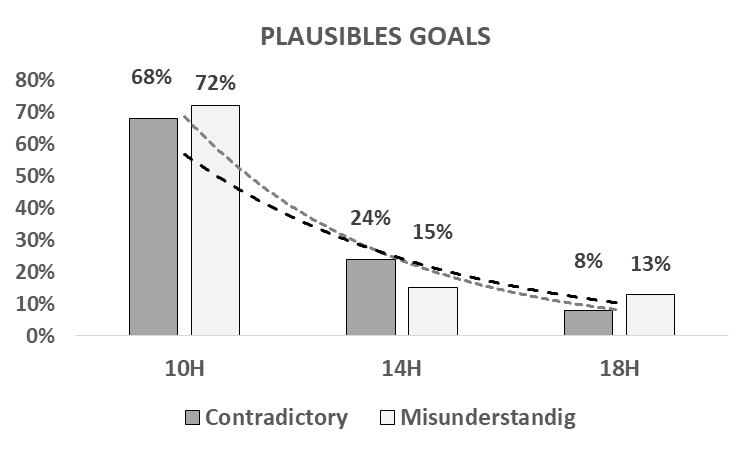


**Figure 2:** Diurnal variation of the elements of RPD model according to contradictory and misunderstanding forms.

| **Sharing modes of the contradictory and misunderstanding forms in Match 1 (10h00) (Figure 3)** | | | | | | | |
| --- | --- | --- | --- | --- | --- | --- | --- |
|  |  |  |  |  |  |  |  |
| Forms | | Match 1 (10h00) | | Match 2 (14h00) | | Match 3 (18h00) | |
|  |  | N | (%) | N | (%) | N | (%) |
| Contradictory | | 10 | (66,66%) | 3 | (20,00%) | 2 | (13,33%) |
| Misunderstanding | | 15 | (65,21%) | 5 | (21,73%) | 3 | (13,04%) |

**Figure 3:** Evolution of sharing mode of the contradictory and misunderstanding forms.
