# Supplementary material for: Influence of diurnal variations on cognitive coordination and misunderstanding in elite male handball players
Source: PeerJ. 2026 Jan 15;14:e20370. doi: 10.7717/peerj.20370 (PMC12812274; doi:10.7717/peerj.20370)
Supplement: Supplemental Information 4 — Verbalized interactions in team play scenarios, focusing on misunderstanding and contradiction as key factors influencing game dynamics. It includes coded sequences of actions, player involvement, and shared or divergent expectations. Systematically organizes the identified forms of misunderstanding and contradiction, contributing to the analysis of decision-making processes in collective sports. [file peerj-14-20370-s004.docx]

**MATCH 1**

| **Attaque** | **Séquences** | **Joueur**  **impliqué** | **Extrait de Verbalisation** | **Nombre de joueurs impliqué** | **Actions** | **Indices** | **Buts plausibles** | **Attentes** | **Contenus typiques partagés** | **Formes typiques déduits** |
| --- | --- | --- | --- | --- | --- | --- | --- | --- | --- | --- |
| **Attaque 1** | **A1.2** | **DC :**  **ALG :** | Je fais un dribble **(A)** et j’avance vers la zone de 6-9m **(A)**, je veux donner la balle à PVT **(AT),** je me trouve bloqué **(I)**, on joue que deux en arrière **(I)**, je tente de faire une passe à ALG **(AT),** je vois ALG est loin de l’action **(I)**, je tourne sur la D **(A)** pour chercher un autre partenaire **(B)**, j’avance **(A)**, alors je fais une passe à ARD **(A)**.  Je vois ARD n’est pas libre **(I)**, il est entouré de quatre défenseurs **(I)**, je recule vers la ligne médiane **(A)** je tente de lui offrir une solution de passe**(AT).**  Je regarde ce qui se passe **(A),** je vois DC est bloqué **(I)**, Je sors de mon aile **(A)**, je tente de lui aider **(AT)**, alors je bouge **(A)** pour lui offrir une solution **(B)**,… DC ne me vois plus **(I),** il donne la balle à ARD **(I).** | 2 | Non-coordonnées | Non- partagés | Divergents | Non-renseignées | C6 | Incompréhension |
|  |  |  |  |  |  |  |  |  |  |  |
| **Attaque 2** | **A2.3** | **DC :**  **ARD :**  **ALD :** | J’ai la balle **(I)**, je m’écarte vers la D **(A)**, je veux donner la balle à ALD (**AT)** mais je me trouve bloqué par deux défenseurs **(I)**, du coup, ARD est près de moi **(I),** j’avance **(A)** et je ne trouve pas une autre solution **(I)** que de lui faire une passe **(A).**  DC s’écarte sur la droite **(I)**, il vise ALD dans son champ visuel **(I)** et du coup je constate qu’il m’exclue de cette action **(I).**  Je reçois une passe inattendue de DC **(I)**, je la passe directement à ALD **(A).** Je recule trois pas **(A)** en attendant la passe d’ALD **(AT)** mais malheureusement ALD me dépasse **(I).**  ARD m’envoie la balle **(I)**, je me trouve marqué par un adversaire **(I)**, je bouge **(A),** je me dirige vers la ligne de 9m **(A)**, pour échanger la balle avec DC **(B)** et puis je lui fais une passe haute **(A).** | 3 | Non-Coordonnées | Non-Partagés | Divergents | Divergentes | C5 | Incompréhension |
|  |  |  |  |  |  |  |  |  |  |  |
| **Attaque 2** | **A3.3** | **DC :**  **ARD :**  **PVT :** | Je pars en course vers la zone de 6-9m **(A)** pour aller au tir **(B)**, je me trouve bloqué par un défenseur **(I)**, je fais une passe à ARD **(A)**.  J’attends une passe d’ARD **(AT),** je mets mes mains en avant **(A)** pour lui offrir une solution de passe **(B)**, du coup, ARD réagit sans réfléchir **(I)**.  On perd la balle **(I).**  On commence le travail avec mes partenaires en coopération et puis chacun d’entre nous veut se montrer  DC m’envoie la balle **(I)**, je fais une feinte à mon adversaire direct **(A)**, je m’écarte sur la G **(A)**, je tente de repasser la balle à DC **(B)**, je me trouve bloqué par deux défenseurs **(I)**, donc je fais une passe à PVT **(A).**  Je pense qu’on trouve une solution rapide **(AT)**, Mais malheureusement, elle n’était pas réussie **(I)**, on perd la balle **(I)**.  Comme d’habitude, je me positionne sur la zone de défense **(A)**, marqué par deux défenseurs **(I)** en attendant une opportunité d’échange avec mes partenaires **(AT)**.  Je suis la circulation de balle en faisant attention aux arrières **(A)** qui pourraient m’envoyer la balle **(AT)**.  Comme je me trouve entouré par trois défenseurs **(I),** je n’ai jamais attendu une passe d’ARD **(I)**. Je ne suis pas disponible **(I)**.  Tout d’un coup, il me fait une passe basse **(I).** Tout simplement, ARD ne sait pas quoi faire. | 3 | Non-Coordonnées | Partagés | Divergentes | Divergentes | C7 | Contradictoire |
|  |  | **ARD :**  **ARG :** | DC m’envoie la balle **(I)**, je fais une feinte à mon adversaire direct **(A)**, je m’écarte sur la G **(A)**, je tente de repasser la balle à DC **(B)**, je me trouve bloqué par deux défenseurs **(I)**, donc je fais une passe à PVT **(A)**  Je pense qu’on trouve une solution rapide **(AT)**, Mais malheureusement, elle n’était pas réussie **(I)**, on perd la balle **(I)**.  Je me prépare à recevoir la balle **(AT)**.  J’attends une passe d’ARD **(AT),** je lève mes mains **(A)** pour attirer son attention **(B)** et lui offrir une solution de passe **(B),** mais en vain**.** J’en ai un peu marre | 2 | Non-coordonnées | Non- partagés | Divergents | Divergentes |  | Incompréhension |
|  |  |  |  |  |  |  |  |  |  |  |
| **Attaque 4** | **A4.1** | **ALD :** | Je récupère la balle **(A)**, j’essaye d’aller au plus vite **(A)** pour réaliser la montée de balle rapide **(B)**, le repli de la défense adverse se fait rapidement **(I)**, du coup, ARD est un peu éloigné **(I)**, je temporise la vitesse **(A)** afin de chercher un partenaire **(B)**, je recule pour faire une passe à ARD **(B)**.  Je donne la balle à ARD **(A)**, je pars en course vers mon aile **(A)** et j’attends qu’il me refait une passe **(AT)** | 2 | Non-Coordonnées | Partagés | Divergents | Divergentes | C1 | Contradictoire |
|  |  | **ARD :** | ALD part en course **(I),** je voudrais partir vite **(AT)** pour lui soutenir **(B)**, mais je lui trouve avancé par rapport à moi **(I)**, du coup, je prévois qu’il aille au tire **(AT),** j’essaye de m’accélérer pour recevoir un ballon **(B)**.  ALD me fait une passe **(I)**, je fais un dribble **(A)**, je m’écarte vers la G **(A)**, je me trouve bloqué par deux défenseurs **(I)**, je vois ARG libre **(I)**, je lui fais une passe **(A).** |  |  |  |  |  |  |  |
|  |  | **ARD :** | ALD part en course **(I),** je voudrais partir vite **(AT)** pour lui soutenir **(B).**  ALD se trouve avancé par rapport à moi **(I)**, du coup, je prévois qu’il aille au tire **(AT)**, quand même j’essaye de m’accélérer pour recevoir un ballon **(B)**  ALD me fait une passe **(I)**, je fais un dribble **(A)**, je m’écarte vers la G **(A)**, je me trouve bloqué par deux défenseurs **(I)**, je vois ARG libre **(I)**, je lui fais une passe **(A).** | 2 | Non-coordonnées | Non- partagés | Non-renseignés | Divergentes |  | Incompréhension |
|  |  | **DC :** | Je voudrais courir vite **(AT)** pour recevoir un ballon **(B).**  J’attends qu’ARD me fait une passe **(AT)**, je m’écarte sur la D **(A)**, je mets mes mains en avant **(A)**, ARD croise sa course devant moi sans aucun regard **(I).** |  |  |  |  |  |  |  |
|  | **A4.2** | **ALG :**  **ARG :** | Je me mets sur mon aile **(A)**, j’attends qu’ARG m’envoie le ballon **(AT)** puisque j’avais assez d’espace sur la ligne de 6m **(I).**  ARG ne me vois pas du tout **(I).**  Je reçois la balle d’ARD **(I)**, je pars en course en avançant vers la ligne de 6m **(A)**. Deux défenseurs se trouvent devant moi **(I)**, ARD m’appelle **(I)**, je dois y aller au tire tout seul **(B)**, je saute **(A)** et je tire **(A)**, pas de but **(I).** | 2 | Non-coordonnées | Non- partagés | Divergents | Divergentes | C6 | Incompréhension |
|  |  | **ARD :**  **ARG :** | J’attends qu’ARG me refait la passe **(AT)**, je mets les mains en l’air **(A).**  ARG joue tout seul **(I)** et il tire **(I).** L’action de tire d’ARG est inattendue**.**  Je reçois la balle d’ARD **(I),** je pars en course en avançant vers la ligne de 6m **(A).**  Deux défenseurs se trouvent devant moi **(I),** ARD m’appelle **(I),** je dois y aller tout seul **(B),** je saute **(A)** et je tire **(A),** pas de but **(I).** | 2 | Non-Coordonnées | Partagés | Divergents | Divergentes |  | Contradictoire |
|  |  |  |  |  |  |  |  |  |  |  |
| **Attaque 5** | **A5.1** | **DC :**  **ARD :**  **PVT :**  **ARG :** | Je reçois la balle d’ARD **(I)**, j’avance vers la ligne de 9m **(A)**  Je trouve un défenseur devant moi **(I)**, j’essaie de lui dépasser pour aller au tire **(B)**, il m’empêche d’avancer **(I).**  L’entraîneur siffle **(I)**  Je vois DC est bloqué par un défenseur **(I)**, j’attends qu’il me me fasse une passe **(AT),** je lève ma main G **(A)** pour avoir une balle **(B)**, mais DC tourne le dos **(I)** et se dirige vers la G **(I)**.  Je suis dans la zone de défense entre deux adversaires **(I)**.  J’attends une passe de DC **(AT)**  J’essaie d’être disponible pour DC **(B)** afin de lui offrir une solution de passe **(B)**, mais il ne me voit pas **(I)**.  L’entraîneur siffle **(I)**.  Je me prépare à recevoir la balle **(AT),** j’ai les bras en avant **(I)**. Je vois que DC cherche à dépasser son adversaire **(I).** Alors, je recule **(A)**. | 4 | Non-coordonnées | Non- partagés | Divergents | Divergentes | C5 | Incompréhension |
|  | **A5.2** | **ARG :**  **PVT :**  **DC :** | Je croise avec DC **(A).** Il me donne la balle **(I)**, je tente de lui redonner **(B)**.  Vu qu’il est un peu reculé **(I)**, je me prépare pour avancer vers la ligne de 6m **(B).**  Je me trouve bloqué par trois défenseurs **(I)**, je fais une feinte pour les dépasser **(A)** et puis je fais une passe basse à PVT **(A)**  Je récupère la balle **(A)** et je passe à DC **(A)** pour recommencer le jeu **(B)**. Puis, je retourne sur la ligne de 6m **(A)**  Je reste sur ma position **(AT)**  Malgré que je me trouve bloqué par trois défenseurs **(I)**, ARG me fait une passe basse **(I)**, je tente de la récupérer **(B)** mais je n’ai pas pu **(I)**. Un adversaire me pousse **(I)**, la balle est perdue **(I).**  Je me prépare à annoncer une combinaison **(B)** mais ARG bouleverse tout le travail **(I)**.  Je croise ma course avec ARG **(A)**, je me place sur la G **(A).**  J’attends une passe d’ARG **(AT)**, je dois me préparer pour lui aider **(B).**  ARG continue de jouer tout seul **(I)**.  La balle est perdue **(I)** | 3 | Non-Coordonnées | Partagés | Divergents | Divergentes | C6 | Contradictoire |
|  |  |  |  |  |  |  |  |  |  |  |
| **Attaque 6** | **A6.2** | **ARD :** | J’ai la balle **(I)**, je tente de faire une passe à DC **(B)**, je vois un défenseur qui se rapproche vers lui **(I)** et du coup, PVT sort de la zone de 6-9m pour me soutenir **(I)**, et je lui fais une passe **(A).**  PVT avance vers la ligne de 6m **(I)**, lorsqu’il prend la position de saut de tir **(I)**, j’attends qu’il aille au tire **(AT)**. PVT me fait une passe imprévue **(I)**.  La balle tombe **(I)**, je la récupère **(A)** et je la passe directement à ALD **(A)**  Je vois ARD entouré de deux défenseurs **(I)**, donc je pars de la zone de défense **(A)** pour lui offrir une solution de passe **(B).**  Je reçois la balle **(I**), je trouve devant moi trois défenseurs **(I)**, donc je lui redonne la balle directement **(A)**  Je lève ma main **(A)** pour attirer l’attention d’ARD **(B)** et qu’il me fasse une passe **(B)**, les défenseurs sont éloignés **(I),** je voudrais aller au tire **(AT)**, personne ne me voit **(I).** | 3 | Non-coordonnées | Non- partagés | Divergents | Divergentes | C5 | Incompréhension |
|  |  | **PVT :** |  |  |  |  |  |  |  |  |
|  |  | **ARG :** |  |  |  |  |  |  |  |  |
|  | **A6.4** | **ARD :** | Je reçois la balle de PVT **(I)**, je tente de la redonner à DC **(B).** Je suis prêt d’aller au tire **(AT)**, je fais une feinte **(A)** pour passer mon adversaire direct **(B)** et je tire de la ligne de 9m **(I)**. | 2 | Non-coordonnées | Partagés | Divergents | Divergentes | C4 | Contradictoire |
|  |  | **DC :** | Là, j’annonce Yougo **(A)**, ARG me passe la balle **(I)**, je dribble **(A).** J’attends **(AT)** que PVT arrive et croise derrière moi **(I),** je lui redonne la balle **(A)**. Je croise avec ARG **(A).**  J’attends une passe d’ARD **(AT)**, alors, je me prépare pour partir en course **(B)** et échanger la balle avec lui **(B)**. Je vois qu’ARD joue seul **(I)** |  |  |  |  |  |  |  |
|  |  | **ARD :**  **ARG :** | Je reçois la balle de PVT **(I)**, je tente de la redonner à DC **(B).** Je suis prêt d’aller au tire **(AT)**, je dribble **(A)**, j’accélère **(A)**, je fais une feinte pour passer mon adversaire direct **(A)** et je tire **(A)** de la ligne de 9m **(I)**.  Aï, aï, aï… je rate ma chance **(I)**  Je vois l’annonce de Yougo **(I),** Je lève mes mains **(A)** je demande la balle **(A)**, personne ne me voit **(I)**, j’en ai marre en attaque.  J’ai toujours le même problème avec ARD, on est en désaccord. | 2 | Non-coordonnées | Non- partagés | Non-renseignés | Divergentes |  | Incompréhension |
|  |  |  |  |  |  |  |  |  |  |  |
| **Attaque 7** | **A7.2** | **ARG**  **DC :** | J’avance vers la zone de 6-9m **(A)**, je trouve devant moi un défenseur qui m’empêche d’arriver à la ligne de 6m **(I)**, donc je dois faire une passe à DC **(B).**  Dans cette action-là, il y a un malentendu avec DC, c’était prévu de jouer Yougo **(I)**, on va recommencer de nouveau l’action.  ARG m’envoie la balle **(I)**, je tente de la récupérer **(AT)** du coup j’ai un cran de retard **(I)** et je rate la balle **(I).** | 2 | Non-Coordonnées | Partagés | Divergents | Non-renseignées | C5 | Contradictoire |
|  |  | **ARD :** | Je récupère la balle d’ARG **(I)**. Je me prépare d’aller au tire **(B)**, alors j’avance **(A).** Je me trouve bloqué par trois adversaires **(I)**, donc je jette la balle sur la G **(A)**. Heureusement, ARG la récupère **(I).** | 2 | Non-coordonnées | Non- partagés | Divergents | Divergentes |  | Incompréhension |
|  |  | **DC :** | ARG m’envoie la balle **(I),** je tente de la récupérer **(AT),** du coup j’ai un cran de retard **(I)** et je rate la balle **(I).** Ici, j’attends une passe d’ARD **(AT),** donc je me prépare pour la recevoir **(AT),** du coup, il jette la balle sans aucune réflexion **(I).** Ça m’étonne. Je n’arrive pas à comprendre mes partenaires d’arrière (ARG, ARD)**.** |  |  |  |  |  |  |  |
| **Attaque 7** | **A7.3** | **ARG :**  **DC :** | Je récupère la balle **(A)**, je dribble **(A)**, ALG est près de moi **(I)**, je lui fais une passe (**A).**  Je me prépare pour une passe d’ARG pour la deuxième fois **(AT)**, j’ai les bras en avant **(I)**, ARG me fait une feinte de passe **(I)**, je me sens qu’il est bloqué **(I).** ARG passe à ALG **(I)**.  Enfin**,** ALG me fait une passe **(I)**, on fait tourner la balle **(A)** avant que j’annonce **(A)** ce qu’on doit jouer en attaque **(I)**. | 2 | Non-Coordonnées | Partagés | Non-renseignés | Divergentes | C3 | Contradictoire |
|  |  | **ARD :**  **ARG :** | Je lève mes mains en haut **(A)** pour attirer l’attention d’ARG **(B)**, mais ARG ne me vois pas **(I)**. Il décide de jouer seul **(I)**.  Je fais tourner la balle avec DC et ARG dans la zone de 9m **(A).**  Je récupère la balle **(A)**, je dribble **(A)**, ALG est près de moi **(I)**, je lui fais une passe (**A).**  ALG ne comprend pas qu’est-ce qu’on va jouer maintenant, j’aimerais bien s’il reste sur son aile tranquillement **(AT),** ça va nous gêner au niveau des déplacements. | 2 | Non-coordonnées | Non- partagés | Divergents | Non-renseignées |  | Incompréhension |
|  | **A7.5** | **PVT :**  **ARD :** | Je vois ARD a la balle **(I)**, je lève ma main **(A)** pour qu’il me fasse une passe **(B)** afin que je puisse aller au tire **(B)**.  ARD passe à ARG **(I)**. Pour la deuxième fois **(I)**, je lève mes deux mains **(A)** pour attirer l’attention d’ARD **(B)**, ARD continue de jouer tout seul **(I)**. ARD rate de marquer **(I)**  Je reçois la balle de DC **(I)**, je m’écarte sur la gauche **(A)**, je fais un dribble en avançant vers ARG **(A)**, je lui fais une passe **(A).** | 2 | Non-coordonnées | Non- partagés | Divergents | Non-renseignées | C6 | Incompréhension |
|  |  |  |  |  |  |  |  |  |  |  |
|  |  |  |  |  |  |  |  |  |  |  |
| **Attaque 8** | **A8.1** | **ARD :**  **ARG :** | Lorsqu’ARG avance vers la ligne de 6m **(I)**, je lève ma main **(A)** pour qu’il m’envoie la balle **(B)**, car je me trouve dans une bonne position **(I)** pour aller au tire **(B)**, ARG ne me vois pas **(I)**  Je reçois le ballon d’ALG **(I),** j’avance vers la zone de 6-9m **(A),** je dribble **(A),** je cherche une position **(A)** pour se pénétrer entre les défenseurs sur la ligne de 6m **(B),** je me trouve bloqué par deux adversaires **(I).** Je tente de faire une passe à DC **(AT),** mais je n’arrive pas **(A).** | 2 | Non-coordonnées | Non- partagés | Divergents | Non-renseignées | C6 | Incompréhension |
|  |  | **DC :**  **ARG :** | Je vois qu’ARG a la balle **(I)** et avance vers la zone de défense **(I)**, je pars en course en même temps **(A)**, j’attends qu’il me fasse une passe **(AT)** pour avancer **(B)** et aller au tire **(B)**, elle est mise en échec par le défenseur **(I)**. Faute (**I)**.  Ici, j’attends que DC s’écarte sur la gauche **(AT)** mais il reste au centre **(I)**, la balle est perdue **(I).** | 2 | Non-Coordonnées | Partagés | Non-renseignés | Divergents |  | Contradictoire |
|  |  |  |  |  |  |  |  |  |  |  |
| **Attaque 9** | **A9.1** | **ARG :**  **DC :** | Je récupère la balle **(A)**, j’essaie de trouver une solution **(B),** je prévois que je puisse aller au tire **(AT)**, je dribble en avançant **(A)** vers la ligne de 6m **(I)**, il y a trois adversaires qui me bloquent **(I)**, je tente d’avancer **(AT)** puis je fais une passe haute à DC **(A).**  Ici, DC reste sur sa position **(I)** au lieu de s’écarter sur la gauche. J’attends le croisement **(AT)**,  Je vois ARG joue très vite sans partage **(I)** comme d’habitude. J’attends une passe de sa part **(AT).** Mais, Il joue seul **(I)**, ça m’étonne vraiment.  ARG se trouve bloqué **(I)**, enfin, il me fait une passe **(I)**, je trouve devant moi quatre adversaires **(I)**, je fais une feinte **(A)** pour passer les adversaires **(B)**, je tourne à droite **(A)**, je passe le ballon à ARD **(A)** | 3 | Non-coordonnées | Non- partagés | Divergents | Divergentes | C7 | Incompréhension |
|  |  |  |  |  |  |  |  |  |  |  |
|  |  | **PVT :** | Je garde une bonne position **(I)** afin d’être disponible pour ARG **(B).** Je prévois avoir une passe d’ARG **(AT)** et lui donner une solution de passe **(B).**  ARG continue de jouer seul **(I).** |  |  |  |  |  |  |  |
|  | **A9.2** | **ALG :**  **ARG :** | Je me mets sur l’aile G **(A).**  Les défenseurs sont éloignés **(I)**, il y a une opportunité de tire **(I)**, j’appelle ARG pas mal de fois **(A)** pour avoir une passe **(B)**, et lui offrir une solution **(B)** mais malheureusement sa position ne lui permet pas de me faire une passe **(I).**  Je reçois la balle de DC **(I)**, je la redonne directement **(A)**, DC me refait la balle **(I).**  Ici, je voudrais faire une passe à ALG **(AT)**, mais comme je me trouve mal placé **(I)**, donc je fais une passe haute à ARD **(A)**. | 2 | Non-Coordonnées | Partagés | Non-renseignés | Divergentes | C3 | Contradictoire |
|  | **A9.3** | **ARD :**  **ARG :**  **DC :** | DC m’envoie la balle **(I)**, je souhaite faire une passe à PVT **(AT)** qui se trouve sur la ligne de 6m **(I)** et comme je me trouve bloqué par deux défenseurs **(I)**, j’envoie la balle à DC **(A).**  J’attends que DC me redonne la balle **(AT).**  Je me prépare pour recevoir une balle de DC **(AT)**, j’avance un pas **(A)** en attendant une passe **(AT),** puis je vois DC avance vers la ligne de 6m vite **(I)**, il se prépare pour le tire **(I).**  ARD m’envoie la balle **(I)**, j’espère aller au tire seul **(AT)**, j’avance **(A)** vers la zone de 6-9m **(I)**, je tente aller au tir **(AT)** alors, je fais une feinte **(A)** pour passer mon adversaire direct **(B)** et je tire **(A)**.  Malheureusement, je rate le but **(I).** | 3 | Non-coordonnées | Non- partagés | Divergents | Divergentes | C5 | Incompréhension |
|  |  |  |  |  |  |  |  |  |  |  |
| **Attaque 10** | **A10.2** | **DC :**  **ARG :**  **ALG :** | Je passe à ARG **(A)**, je souhaite qu’il me repasse la balle **(AT)**, je vois qu’ARG joue sans aucune coordination avec moi **(I)**, le même problème toujours avec lui, je reste au centre sans bouger **(AT).**  DC me donne la balle **(I)**, en ce moment-là, je me prépare pour traverser le bloc de la défense **(B)**, je me trouve bloqué par deux défenseurs **(I)**, il n’y a pas d’espace **(I)** pour y aller tirer **(B)**, je voudrais faire une passe à PVT **(AT).**  Quand je vois ARG avance avec la balle **(I)**, je me prépare pour avoir une passe **(AT).** ARG ne me vois pas **(I).**  Je me trouve seul **(I)**, il y a de l’espace pour tirer d’ici **(I).** | 3 | Non-coordonnées | Non- partagés | Divergents | Divergentes | C7 | Incompréhension |
|  |  | **PVT :** | ARG m’envoie la balle **(I)** malgré que je ne suis pas disponible **(I)**, je tente de récupérer la balle **(B)**, le défenseur me pousse dans le dos **(I)**, la balle tombe **(I)**, c’est une faute **(I).**  Il n’y a pas d’espace **(I)** pour y aller tirer **(B),** je voudrais faire une passe à PVT **(AT).** | 2 | Non-Coordonnées | Partagés | Divergent | Divergentes |  | Contradictoire |
|  |  | **ARG :** |  |  |  |  |  |  |  |  |

| Formes | | modèle RPD | | | |
| --- | --- | --- | --- | --- | --- |
|  |  | Actions (A) | Indices pertinents (I) | Buts (B) | Attentes (AT) |
| Contradictoire | 10 | 44 | 71 | 26 | 19 |
| Incompréhension | 15 | 74 | 111 | 34 | 39 |

**MATCH 2**

| **Attaque** | **Séquences** | **Joueur impliqué** | **Extrait de Verbalisation** | **Nombre de joueurs impliqué** | **Actions** | **Indices** | **Buts plausibles** | **Attentes** | **Contenus typiques partagés** | **Formes typiques déduits** |
| --- | --- | --- | --- | --- | --- | --- | --- | --- | --- | --- |
|  |  |  |  |  |  |  |  |  |  |  |
| **Attaque 2** | **A2.1** | **DC :** | PVT récupère la balle **(I)** et me fait une passe **(I)**, je pars en course vers l’avant **(A)**, je passe à ARD **(A)** qui la repasse à ALD **(I)**, et puis il me redonne la balle **(I)**, on fait circuler la balle **(A)** et je me prépare d’annoncer une combinaison **(I).**  Ici, j’annonce Yougo **(A)** avec ma main presque trois fois **(I)**, mais PVT ne me voit pas **(I)**, son regard est focalisé sur une autre chose **(I),** je tente trouver une solution **(AT)** | 2 | Non-coordonnées | Non- partagés | Divergents | Divergentes | C3 | Incompréhension |
|  |  | **PVT :** | Je pars vite **(A)** vers la zone de défense **(I)** pour me placer près de mes défenseurs **(B)** et je garde ma position **(I).**  J’attends l’annonce de DC **(AT).** |  |  |  |  |  |  |  |
|  | **A2.2** | **DC :**  **PVT :** | J’annonce de nouveau la combinaison Yougo **(A)** avec ma main et ma voix **(I)** pour attirer l’attention de PVT **(B)** afin de faciliter l’échange de la balle **(B).**  Je vois PVT bouge **(I)**  Je me prépare **(A)** pour lancer le ballon à PVT **(B)** et puis à croiser la course avec ARG **(B)**  Je vois l’annonce **(I)** et j’entends le mot ‘prochainement’ de DC **(I)**, puis il lève sa main **(I)**  Je pars en course **(A)** vers la zone d’attaque **(I)**, je croise derrière DC **(A)**, j’attends la balle **(AT)**, j’ai un cran de retard **(I)** et du coup la balle tombe **(I)**.  Je n’arrive pas à la rattraper avec mes mains **(A)**, j’ai essayé **(A)** je recule vers la ligne médiane **(A)** pour la récupérer **(B)**, un défenseur me pousse dans le dos **(I).**Je tombe **(A)** | 2 | Non-Coordonnées | Partagés | Convergents | Non-renseignées | C4 | Contradictoire |
|  | **A2.3** | **DC :**  **ARG :** | Je récupère la balle **(A)**, je me mets à dribbler **(A)** j’avance **(A)** vers la ligne de 6m **(I)**, et hop je fais une passe à ARG **(A)** qui garde une bonne position **(I)** pour aller vite **(B)**, je ne sais pas qu’est-ce qu’il a, il n’arrive pas à rattraper la balle **(I).**  Je vois que DC a la balle **(I)**, je dois me préparer **(A)** pour lui offrir une solution de passe **(B)**, j’avance en course **(A)** pour attirer son attention **(B)**, il me lance la balle trop derrière **(I)**, j’essaye de la rattraper **(A),** du coup, j’ai un cran de retard **(I),** je rate la balle **(I).** | 2 | Non-Coordonnées | Partagés | Non-renseignés | Non-renseignées | C5 | Contradictoire |
|  |  |  |  |  |  |  |  |  |  |  |
| **Attaque 3** | **A3.3** | **DC :**  **ARD :** | Je reçois la balle **(I)** et j’avance rapidement **(A),** je dribble **(A)**, hop, il y a un défenseur devant moi **(I)**, mon but c’est de lui dépasser **(B)**, donc je fais une feinte **(A)** et je me reste prudent **(I).**  J’avance vers la ligne de 6m **(I)** et je tire **(A)**.  Malheureusement, je rate complètement mon tire **(I)**  Lorsque DC avance **(I)**, je croise derrière lui **(A)**, j’attends qu’il me fasse une passe **(AT**) pour aller au tire **(B).**  J’aurai aimé terminer l’attaque vers l’extérieur. | 2 | Non-coordonnées | Non- partagés | Non-renseignés | Divergentes | C5 | Incompréhension |
|  |  |  |  |  |  |  |  |  |  |  |
| **Attaque 6** | **A6.1** | **PVT :**  **ARD :** | Je rentre vite dans la zone de défense **(A)** avant le repli de défenseurs **(I)** et je garde ma position **(I)** pour offrir une solution de passe à mes partenaires **(B)**  Je mets les bras en l’air **(A)** pour attirer l’attention d’ARD **(B)**, ARD ne me voit pas **(I)**.  Ben là, je pars très vite **(A)** pour me replacer en avant **(B)** et je demande une passe de DC **(A)**, il m’envoie la balle **(I)**  Je vois DC avance **(I).** | 2 | Non-coordonnées | Non- partagés | Divergents | Non-renseignées | C1 | Incompréhension |
|  | **A6.2** | **DC :**  **ARG :** | ARD me donne la balle **(I),** je me trouve gêné par deux défenseurs **(I),** je voudrais faire une passe à PVT **(AT)**, je jette la balle à PVT **(A)**  Oh là là…une faute **(I)**  Quand DC avance avec le ballon **(I)**, j’avance en parallèle avec lui **(A)**, alors je me prépare pour avoir une passe de DC **(AT)**.  Faute **(I).** | 2 | Non-coordonnées | Non- partagés | Non-renseignés | Divergentes | C6 | Incompréhension |
|  |  |  |  |  |  |  |  |  |  |  |
| **Attaque 9** | **A9.2** | **DC :**  **PVT :** | ARG me fait une passe **(I)**, j’avance **(A)** vers la ligne de 6m **(I)** et je dribble **(A)**, je me trouve entouré de trois défenseurs **(I)**, je cherche un partenaire **(A)** et là je jette la balle à PVT **(A)** pour avancer **(B).**  Je vois DC avance **(I)** avec la balle **(I)**, je me prépare pour avoir une passe **(AT)**, et comme je me trouve bloqué **(I)**, je n’arrive pas à récupérer la balle **(I)**, il me la lance **(I)**, un des adversaires me pousse **(I)**, on perd le ballon **(I).** | 2 | Non-Coordonnées | Partagés | Non-renseignés | Divergentes | C5 | Contradictoire |
|  |  | **ARD :** | J’attends que DC me fasse une passe **(AT)** mais il ne me voit pas **(I)**, je vois qu’il joue seul **(I)**  La passe à PVT est incorrecte **(I),** il fallait que DC fasse une passe à ARG pour qu’il puisse avancer avec la balle **(B)**  Vers la ligne de 6m **(I)** je me trouve entouré de trois défenseurs **(I)**, je jette la balle à PVT **(A)**  Je fais une passe simple à DC **(A)**  J’attends qu’il me refait la passe **(AT)**  DC réagit vite ici **(I).** | 3 | Non-coordonnées | Non- partagés | Non-renseignés | Divergentes |  | Incompréhension |
|  |  | **DC :** |  |  |  |  |  |  |  |  |
|  |  | **ARG :** |  |  |  |  |  |  |  |  |

| Formes | | modèle RPD | | | |
| --- | --- | --- | --- | --- | --- |
|  |  | Actions (A) | Indices pertinents (I) | Buts (B) | Attentes (AT) |
| Contradictoire | 3 | 19 | 26 | 9 | 2 |
| Incompréhension | 5 | 17 | 32 | 7 | 7 |

**MATCH 3**

| **Attaque** | **Séquence** | **Joueur impliqué** | **Extrait de Verbalisation** | **Nombre de joueurs impliqué** | **Actions** | **Indices** | **Buts plausibles** | **Attentes** | **Contenus typiques partagés** | **Formes typiques déduits** |
| --- | --- | --- | --- | --- | --- | --- | --- | --- | --- | --- |
| **Attaque 1** | **A1.4** | **ALD**  **DC :**  **ARD** **:** | J’attends une passe de DC **(AT)** et puis d’ARD **(AT)** mais personne ne me voit **(I)**, donc je reste sur mon aile tranquille **(I).**  ARG me refait la passe **(I)**, je fais un petit tour sur la gauche **(A)**, j’avance vers la ligne de 6m **(A)**, pour aller au tir **(B)** je vois un trou entre deux défenseurs **(I)**, hop je fais une passe à PVT **(A)**  Je recule vers la zone de 9m **(A)** et après j’avance avec DC **(A)** | 3 | Non-coordonnées | Non- partagés | Non-renseignés | Divergentes | C6 | Incompréhension |
|  |  |  |  |  |  |  |  |  |  |  |
| **Attaque 5** |  | **DC :**  **ARD :**  **ARG :** | Je reçois la balle d’ARD **(I)**, je cours **(A)** vers la zone de défense **(I)**, j’essaie de trouver de solutions **(B)** et aller au tire **(B)**, je trouve deux adversaires devant moi **(I)**, la défense me gêne d’avancer **(I)**.  ALD ne bouge plus **(I)**, je n’ai pas trouvé une solution **(I)** que de faire une passe arrière **(A)**, je jette la balle **(A)**  Ici, je vois DC avance **(I)**, alors je me prépare pour avoir un futur ballon **(AT),** du coup DC fait une passe en arrière **(I)**.  L’entraîneur toujours nous déconseille de ne pas faire cette passe, je récupère le ballon difficilement avec la main gauche **(A),** en ce moment-là, je ne sais pas quoi faire exactement  Je fais une passe à ARG **(A)**  Lorsqu’ARD reçoit la balle **(I)**, je pense qu’il va avancer vers la ligne de 6m **(AT)**, donc il n’y aura plus une opportunité de passe **(I)**  Du coup, je reçois une passe inattendue **(I)**, j’avance **(A)**, je trouve devant moi un bloc de défenseurs **(I)**, je suis stoppé par deux adversaires **(I)**, je tente de faire une passe **(B),** je tombe **(A)** | 3 | Non-coordonnées | Non- partagés | Divergents | Divergentes | C7 | Incompréhension |
|  | **A5.3** |  |  |  |  |  |  |  |  |  |
|  |  |  |  |  |  |  |  |  |  |  |
| **Attaque 6** |  | **ARD :**  **DC**  **PVT :** | Je fais un dribble **(A)** et j’avance **(A)**, je trouve devant moi un adversaire **(I)** qui me bloque **(I)**, j’essaye de faire une passe à DC **(B)**, malheureusement, un adversaire intercepte la balle **(I)**  ARD fait un dribble **(I)**, il me fait une passe **(I)**, un adversaire l’intercepte **(I)**, on perd la balle **(I)**  ARD toujours fait un seul dribble, d’où il se trouve obligé de réaliser une passe immédiatement, ouf son travail m’énerve beaucoup et comme je suis le meneur de jeu, je lui conseille ‘pas mal de fois’ d’éviter cette méthode, car ça nous fait perdre la balle, mais malheureusement, comme-ci je ne l’ai rien dit.  ARD avance vers la zone de 6-9m **(I)**, je me prépare pour lui offrir une solution de passe **(B)**, je vois qu’il passe la balle en arrière **(I)**  J’aurai aimé qu’il me fasse cette passe, car j’avais l’opportunité d’y aller au tire  Oh, on perd la balle ! **(I)** | 3 | Non-coordonnées | Non- partagés | Divergents | Non-renseignées | C6 | Incompréhension |
|  | **A6.3** |  |  |  |  |  |  |  |  |  |
|  |  |  |  |  |  |  |  |  |  |  |
|  |  |  |  |  |  |  |  |  |  |  |
| **Attaque 7** | **A7.3** | **ARG :**  **PVT :** | ALG me repasse la balle **(I)**, alors j’avance vers la ligne de 6m **(A)**.  Je trouve deux défenseurs m’empêchent d’avancer **(I)**, j’attends que PVT intervient **(AT)**, pour me soutenir **(B)** l’un de deux adversaires me pousse **(I)**, je tombe **(A)**.  Je suis le déroulement du jeu **(A)**  ARG avance vers la ligne de 6m **(I)**, je ne sais pas quoi faire exactement.  Je pense qu’ARG termine l’action tout seul **(AT)** | 2 | Non-coordonnées | Partagés | Divergents | Divergentes | C7 | Contradictoire |
|  |  |  |  |  |  |  |  |  |  |  |
| **Attaque 9** | **A9.3** | **DC :**    **ARG :** | J’envoie la balle à ARG **(A).**  J’avance avec ARG **(A)** pour lui fournir une solution de passe en avant **(B)**  Je reçois la balle de DC **(I)**.  J’avance rapidement vers le centre **(A)**, je dribble **(A)** pour aller au tire **(B)**, je me trouve bloqué par des défenseurs qui sont regroupés **(I)**, la balle est perdue | 2 | Non-Coordonnées | Partagés | Divergents | Divergentes | C7 | Contradictoire |
|  |  |  |  |  |  |  |  |  |  |  |
|  |  |  |  |  |  |  |  |  |  |  |

| Formes | | modèle RPD | | | |
| --- | --- | --- | --- | --- | --- |
|  |  | Actions (A) | Indices pertinents (I) | Buts (B) | Attentes (AT) |
| Contradictoire | 2 | 7 | 6 | 3 | 2 |
| Incompréhension | 3 | 14 | 27 | 6 | 4 |
